# LINT, a Novel dL(3)mbt-Containing Complex, Represses Malignant Brain Tumour Signature Genes

**DOI:** 10.1371/journal.pgen.1002676

**Published:** 2012-05-03

**Authors:** Karin Meier, Eve-Lyne Mathieu, Florian Finkernagel, L. Maximilian Reuter, Maren Scharfe, Gunther Doehlemann, Michael Jarek, Alexander Brehm

**Affiliations:** 1 Institut für Molekularbiologie und Tumorforschung, Philipps-Universität Marburg, Marburg, Germany; 2 Helmholtz Center for Infection Research, Braunschweig, Germany; 3 Max-Planck-Institut für Terrestrische Mikrobiologie, Marburg, Germany; Max-Planck Institute of Immunobiology, Germany

## Abstract

Mutations in the *l(3)mbt* tumour suppressor result in overproliferation of *Drosophila* larval brains. Recently, the derepression of different gene classes in *l(3)*mbt mutants was shown to be causal for transformation. However, the molecular mechanisms of dL(3)mbt-mediated gene repression are not understood. Here, we identify LINT, the major dL(3)mbt complex of *Drosophila*. LINT has three core subunits—dL(3)mbt, dCoREST, and dLint-1—and is expressed in cell lines, embryos, and larval brain. Using genome-wide ChIP–Seq analysis, we show that dLint-1 binds close to the TSS of tumour-relevant target genes. Depletion of the LINT core subunits results in derepression of these genes. By contrast, histone deacetylase, histone methylase, and histone demethylase activities are not required to maintain repression. Our results support a direct role of LINT in the repression of brain tumour-relevant target genes by restricting promoter access.

## Author Summary

Mutations in the *l(3)mbt* result in the formation of brain tumours. The molecular basis underlying this phenotype has remained obscure. Here, we have isolated LINT, a novel protein complex containing dL(3)mbt, the corepressor dCoREST, and the uncharacterised protein dLint-1. We have used genome-wide ChIP–Seq analysis to map the binding sites of LINT. LINT occupies the promoters of many genes that are deregulated in *l(3)mbt* brain tumours, suggesting that these genes are repressed by LINT. Indeed, RNAi–mediated depletion of LINT subunits results in the derepression of these genes. Surprisingly, LINT-mediated repression is largely independent of histone modification status, arguing for a repression mechanism that operates by restricting promoter access.

## Introduction

Regulation of chromatin structure by enzymatic and non-enzymatic mechanisms plays a pivotal role in the proliferation, differentiation and transformation of cells. Components of histone modifying protein complexes and ATP-dependent chromatin remodelers are misexpressed or mutated in cancer and other diseases [1]. While a connection between defective chromatin regulation and disease is well established in numerous cases, the nature and mechanisms of action of the protein complexes involved are not well understood.

A temperature sensitive mutation in the *Drosophila* gene *l(3)mbt* results in aberrant overproliferation of cells in the brains of third instar larvae [2]. This generates malignant brain tumours with the potential to metastasize. Two recent studies have identified genes that are misexpressed in *l(3)mbt^ts^* brain tumours [3], [4]. *l(3)mbt* inactivation results in the specific deregulation of 102 genes that constitute the malignant brain tumour signature (MBTS) [3]. 32 MBTS genes encode proteins important for germ line function and mutation of some of these genes rescues the *l(3)mbt^ts^* phenotype [3]. It was also reported that a group of 7 genes that are targeted by the Salvador-Warts-Hippo (SWH) signaling pathway are derepressed in brain tumour tissue and forced overexpression of some of these genes replicates the *l(3)mbt^ts^* phenotype [4]. dL(3)mbt protein binds many of the MBTS germline and SWH target genes suggesting that these genes are direct targets of dL(3)mbt [4].

L3MBTL1 is the closest human homolog of dL(3)mbt. The MBT domains of L3MBTL1 compact nucleosomes bearing H4K20me1 or H1bK26me1 modifications *in vitro* [5]. L3MBTL1 chromatin association and L3MBTL1-mediated repression depend to a large degree on H4K20 methylation and the H4K20-specific histone methylase PR-SET7 [6]. L3MBTL1 is part of a complex containing pRb, HP1γ, H1b and core histones which has been suggested to repress transcription by increasing nucleosome compaction at target genes [5]. The individual contributions of L3MBTL1 complex subunits to repression are not clear.

*Drosophila* L(3)mbt associates with the MybMuvB/dREAM complex at substoichiometric levels [7]. Similar to the L3MBTL1 complex, MybMuvB/dREAM contains pRb proteins but lacks HP1 and histone proteins [7], [8]. Mutations in core components of the MybMuvB/dREAM complex do not give rise to larval brain tumours raising the possibility that repression of tumour-relevant genes is maintained by a different dL(3)mbt complex.

Here, we use immunoaffinity and conventional chromatography to purify LINT, the major dL(3)mbt complex in *Drosophila*. LINT is composed of three subunits: dL(3)mbt, dCoREST and the uncharacterised protein dLint-1. LINT is expressed in cell lines, embryos and larval brains. Indirect immunofluorescence analysis of larval polytene chromosomes revealed LINT subunit colocalisation at many interbands. In agreement with this finding, genomewide ChIP-Seq demonstrated that dL(3)mbt and dLint-1 bind together in the vicinity of the transcriptional start site (TSS) of many genes. RNAi-mediated depletion of LINT derepresses MBTS germline genes in both cell lines and in larvae. By contrast, SWH target genes are not derepressed in LINT depleted cells. Repression of MBTS germline genes depends on the presence of all three core LINT subunits at the promoter but is not affected by depletion of dLsd1, dRpd3, dPR-Set7 and other histone modifying enzymes suggesting that LINT represses germline-specific genes by binding to their promoters and by restricting access of transcription factors and RNA polymerase II. Our study identifies the novel LINT complex as an important repressor of germline-specific genes that are derepressed in malignant brain tumours in *Drosophila*.

## Results

### dLint-1 is a novel dL(3)mbt-interacting protein

**Figure 1:**
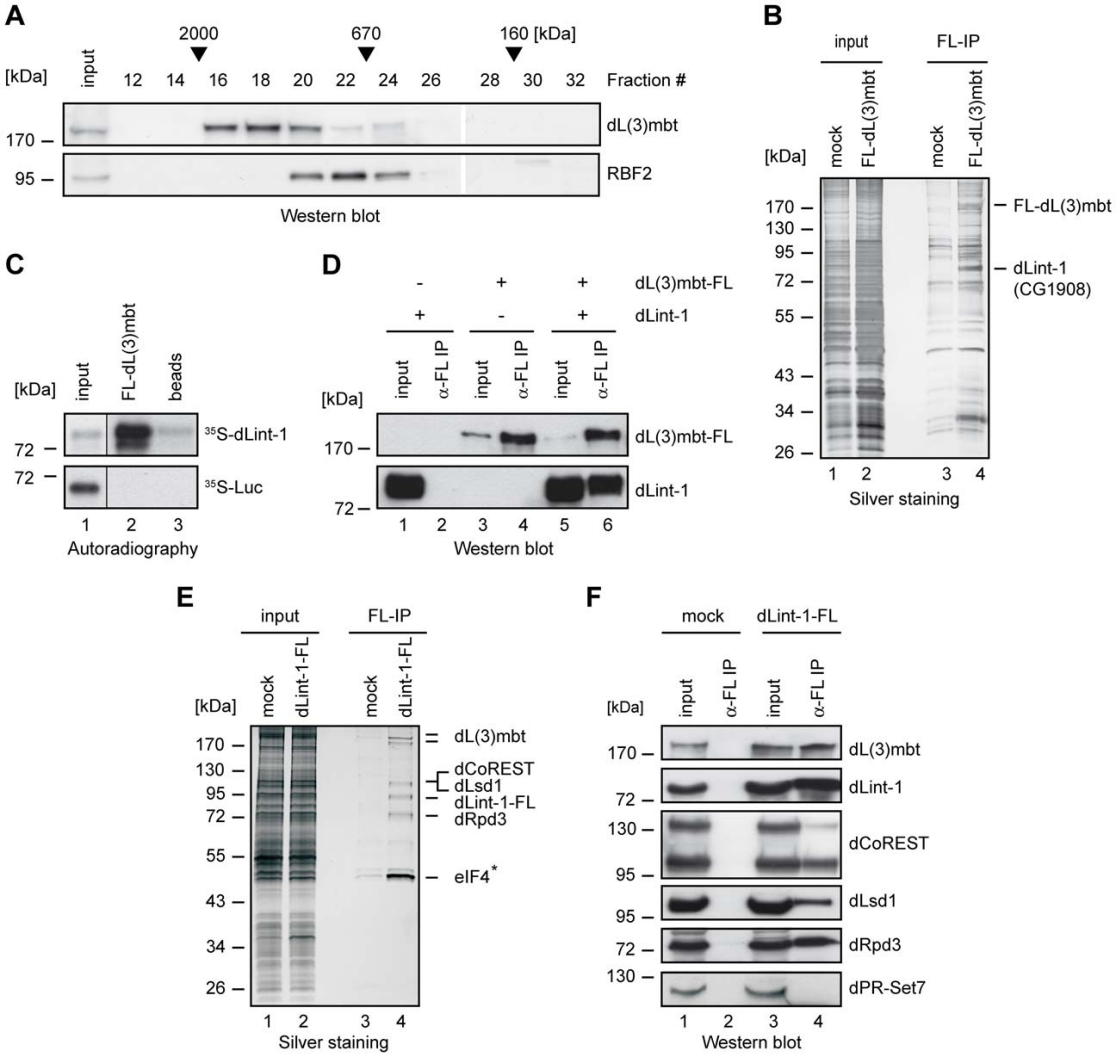
dLint-1 is a novel dL(3)mbt–interacting protein. (A) Nuclear extract from Kc cells was fractionated over a Superose 6 column. Fractions were analyzed by Western blot using the antibodies indicated. Fraction numbers and molecular mass standards are denoted on top. Input: 5% of extract loaded. (B) Nuclear extracts from control S2 cells (mock, lanes 1 and 3) and S2 cells stably expressing FLAG-dL(3)mbt (lanes 2 and 4) were subjected to FLAG affinity purification, elution with FLAG peptide, SDS-PAGE and silver staining (lanes 3 and 4). Input: 2 µg of nuclear extracts (lanes 1 and 2). The positions of FLAG-dL(3)mbt and dLint-1 are indicated on the right. (C) *In vitro* translated, ^35^S-labeled dLint-1 (upper panel) or luciferase (lower panel) were incubated with FLAG beads (beads, lane 3) or beads loaded with FLAG-dL(3)mbt (lane 2). Bound proteins were separated by SDS-PAGE and detected by autoradiography. Lane 1: 1% input. (D) Sf9 cells were infected with baculoviruses expressing dL(3)mbt-FLAG or dLint-1 as indicated on top. Extracts were immunoprecipitated and subjected to Western blot using FLAG and dLint-1 #2 antibodies (lanes 2, 4 and 6). Lanes 1, 3 and 5: 5% input. (E) Nuclear extracts from control S2 cells (mock, lanes 1 and 3) and S2 cells stably expressing dLint-1-FLAG (lanes 2 and 4) were subjected to FLAG affinity purification, elution with FLAG peptide, SDS-PAGE and silver staining (lanes 3 and 4). Input: 2 µg of nuclear extracts (lanes 1 and 2). The position of dLint-1-FLAG and copurifying proteins are indicated on the right. * denotes that eIF-4B was also recovered from the control and is considered to be a contaminant. Note that dCoREST and dLsd1 have the same molecular weight and comigrate. (F) Nuclear extracts from control S2 cells (mock, lanes 1 and 2) and S2 cells stably expressing FLAG-dLint-1 (lanes 3 and 4) were precipitated with FLAG antibody and analyzed by Western blot as indicated (lanes 2 and 4). dPR-Set7 served as a negative control. Lanes 1 and 3: 5% input.

We fractionated nuclear extracts of Kc cells by gel filtration to assess whether dL(3)mbt is a part of protein complexes. dL(3)mbt eluted in fractions with an apparent molecular weight of 0.67–2.0 MDa (Figure 1A). The bulk of dL(3)mbt separated from the dREAM subunit RBF2 suggesting that dL(3)mbt exists in high molecular weight complexes that are distinct from dREAM.

We established a S2 cell line stably expressing FLAG-tagged dL(3)mbt to facilitate the identification of interacting proteins (Figure S1A). Next, nuclear extracts were bound to FLAG immunoaffinity resin, eluted with FLAG peptide and analyzed by SDS-PAGE (Figure 1B). As a control, nuclear extract from cells not expressing FLAG-tagged dL(3)mbt were processed in parallel. Many polypeptides that were present in both purifications were visualized by silver staining (Figure 1B, compare lanes 3 and 4). Therefore, we consider the majority of these polypeptides to be contaminants. However, two polypeptides of 185 kDa and 85 kDa, respectively, were specifically purified from FLAG-dL(3)mbt expressing cells. The 185 kDa polypeptide was identified by peptide mass fingerprinting as dL(3)mbt (Figure S1B). The 85 kDa polypeptide was identified as the gene product of *CG1908*, a protein of unknown function. From hereon, we will refer to this protein as dLint-1 (*Drosophila L*(3)mbt *int*eracting protein *1*). Sequence analysis identified a cysteine-rich region near the C-terminus of dLint-1 with similarities to a PHD finger (Figure S2).

We carried out *in vitro* interaction assays to verify that recombinant dL(3)mbt and dLint-1 interact in a robust manner (Figure 1C and 1D). FLAG-tagged dL(3)mbt bound specifically to *in vitro* translated dLint-1 (Figure 1C). Furthermore, both proteins coimmunoprecipitated from extracts of Sf9 cells infected with recombinant baculoviruses (Figure 1D).

Next, we established an S2 line stably expressing FLAG-tagged dLint-1 and used FLAG immunoaffinity purification to identify dLint-1 interaction partners (Figure S1C). Several polypeptides copurified with dLint-1 (Figure 1E). Peptide fingerprinting identified these as dL(3)mbt, the histone demethylase dLsd1, the corepressor dCoREST and the histone deacetylase dRpd3 (Figure S1D). The identity of these proteins was verified by Western blot (Figure 1F). Three alternative splice forms of dCoREST exist, two of which - a 95 kDa and a 130 kDa polypeptide - are recognized by the antibody we have used [9]. Comparison of signal intensities between isoforms revealed that dLint-1 associated predominantly with the 95 kDa polypeptide.

**Figure 2:**
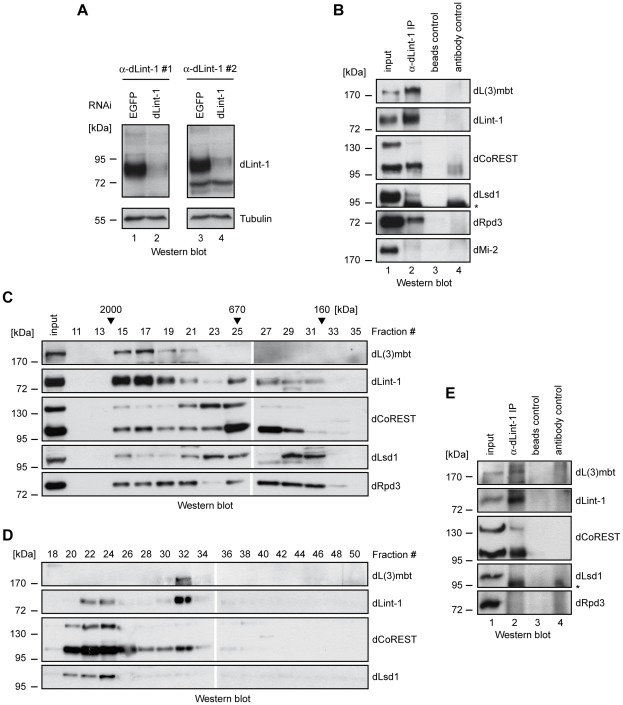
Purification of the LINT complex. (A) Nuclear extracts of Kc cells treated with dsRNA directed against EGFP (control, lanes 1 and 3) and dLint-1 (lanes 2 and 4) were subjected to Western blot using dLint-1 antibody #1 (left upper panel), dLint-1 antibody #2 (right upper panel) and tubulin antibody (lower panels). (B) Nuclear extracts from Kc cells were precipitated with protein G beads (beads control, lane 3) and beads loaded with dLint-1 #1 antibody (lane 2) and analyzed by Western blot as indicated (lanes 2 and 3). dMi-2 served as a negative control. Lane 1: 5% input; lane 4 contains dLint-1 antibody (antibody control). * denotes a polypeptide that unspecifically crossreacts with the dLsd1 antibody (compare lanes 2 and 4). (C) Nuclear extract from Kc cells was fractionated over a Superose 6 column. Fractions were analyzed by Western blot. Fraction numbers and molecular mass standards are denoted on top. Input: 5% of extract loaded. (D) Kc nuclear extract was separated by sequential ion exchange chromatography over Q-Sepharose and MonoQ columns. MonoQ fractions were analyzed by Western blot as indicated. Fraction numbers are denoted on top. (E) Extract from third instar larval brains was precipitated with protein G beads (beads control, lane 3) and beads loaded with dLint-1 antibody (lane 2) and analyzed by Western blot as indicated (lanes 2 and 3). Lane 1: 5% input; lane 4 contains dLint-1 antibody (antibody control). * denotes a polypeptide that unspecifically crossreacts with the dLsd1 antibody (compare lanes 2 and 4).

We raised two dLint-1-specific antisera to characterize the endogenous dLint-1 protein. Both antisera recognized an 80 kDa polypeptide in a Western blot analysis of Kc nuclear extract (Figure 2A). Treatment of Kc cells with double stranded RNA directed against the dLint-1 mRNA greatly decreased the intensity of these bands demonstrating that both antisera are specific for dLint-1. We used dLint-1 antiserum to immunoprecipitate nuclear extracts. Coprecipitation of dL(3)mbt, dLsd1, the 95 kDa isoform of dCoREST and dRpd3 confirmed that these proteins interacted with endogenous dLint-1 in nuclear extracts derived from both cell lines and embryos (Figure 2B, Figure S3).

### Identification of the LINT complex

Endogenous dLint-1 and the 95 kDa dCoREST isoform coeluted with dL(3)mbt in high molecular weight gel filtration fractions (Figure 2C, fractions 15–21). dLsd1 and dRpd3 were detectable in the same fractions. However, unlike dL(3)mbt, dLint-1 and dCoREST, these proteins did not peak in fraction 17. Strong dLint-1, dLsd1, dCoREST and dRpd3 signals were apparent in fractions 23 to 31 (670 kDa to 160 kDa). However, we failed to detect dL(3)mbt in these fractions. The Superose 6 elution profiles are consistent with the presence of dL(3)mbt, dLint-1 and dCoREST in a high molecular weight complex. In addition, one or more additional dLint-1 containing complexes with smaller apparent molecular weight appear to exist.

We used ion exchange chromatography to separate different dLint-1-containing complexes (Figure 2D). Sequential fractionation of nuclear extract over Q-Sepharose and MonoQ columns separated two pools of dLint-1 eluting in different MonoQ fractions. dLint-1 coeluted with dLsd1 and dCoREST in fractions 22 to 24. These fractions did not contain detectable levels of dL(3)mbt. A second pool of dLint-1 coeluted with dL(3)mbt and dCoREST in fraction 32. This fraction did not contain significant amounts of dLsd1.

The elution profiles from both gel filtration and ion exchange columns suggest that dCoREST and dLsd1 polypeptides likely occur in multiple protein complexes. dLint-1, however, appears to be restricted to two separate complexes, one containing dCoREST and dLsd1, the other containing dCoREST and dL(3)mbt. In contrast, dL(3)mbt appears to exist exclusively in a single complex together with dLint-1 and dCoREST. We focused our analysis on the dL(3)mbt/dLint-1/dCoREST complex which we will refer to from hereon as the LINT (dL(3)mbt interacting protein) complex.

dL(3)mbt mutant larvae develop brain tumours at the third instar stage. To determine if LINT is present in this tissue we prepared extracts from larval brains. Immunoprecipitation with dLint-1 antibody resulted in the coprecipitation of dL(3)mbt and dCoREST (Figure 2E). Although dLsd1 and dRpd3 were present in these extracts they failed to coprecipitate. We conclude that third instar larval brains contain the LINT complex. Whether other dLint-1 containing complexes exist in this tissue is unclear.

### dL(3)mbt and dLint-1 colocalise on polytene chromosomes

**Figure 3:**
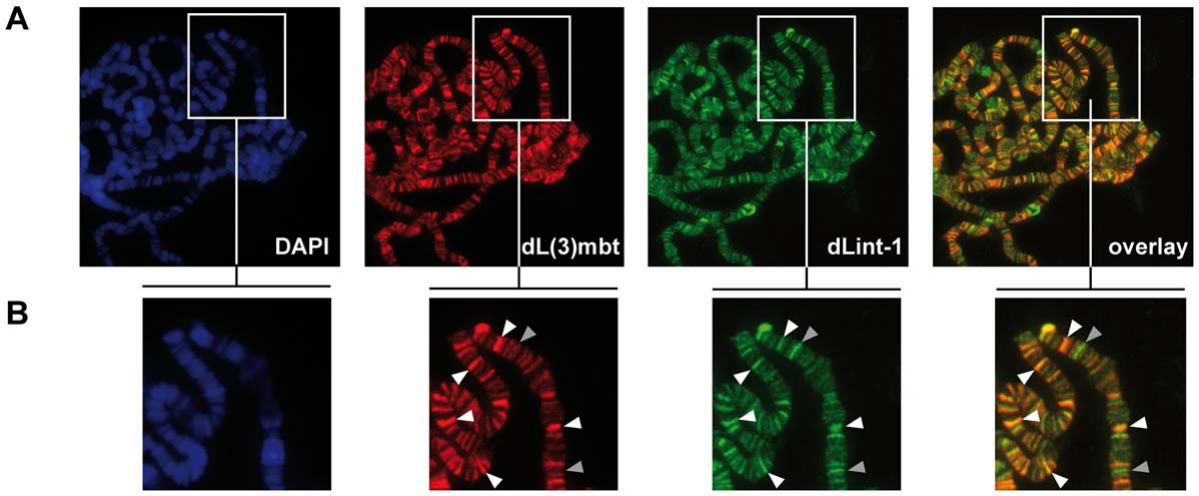
dL(3)mbt and dLint-1 colocalise on polytene chromosomes. (A) Immunofluorescence stainings of polytene chromosomes. Flies carrying an dL(3)mbt transgene under control of UAS were crossed with a salivary gland-specific *sgs58AB*-*GAL4* driver strain. Polytene chromosomes were stained with dL(3)mbt, dLint-1 antibodies and DAPI as indicated. The right panel shows an overlay of the dL(3)mbt and dLint-1 staining. (B) Magnified sections of the panels shown in (A). White and grey arrow heads denote selected prominent sites of colocalization (white) and exclusive dLint-1 binding sites (grey), respectively.

We next asked if subunits of LINT bind chromatin and colocalise at the same regions. Our dL(3)mbt antibody failed to detect endogenous dL(3)mbt when we analyzed polytene chromosomes by indirect immunofluorescence (*data not shown*). However, overexpression of dL(3)mbt in transgenic larvae allowed the detection of numerous interbands occupied by recombinant dL(3)mbt (Figure 3). Costaining with dLint-1 antibody revealed extensive colocalisation between both proteins. We visually assessed 466 bands for dL(3)mbt and dLint-1 binding (see Methods). 83% of these were stained by both antibodies, 12% showed dLint-1 but no or very weak dL(3)mbt binding and 5% appeared to be bound by dL(3)mbt only. These results show that dL(3)mbt and dLint-1 can cooccupy many chromatin regions on polytene chromosomes suggesting that these two proteins not only associate in soluble nuclear extracts but also when bound to chromatin.

### LINT binds to genes deregulated in *l(3)mbt^ts^* brain tumours

**Figure 4:**
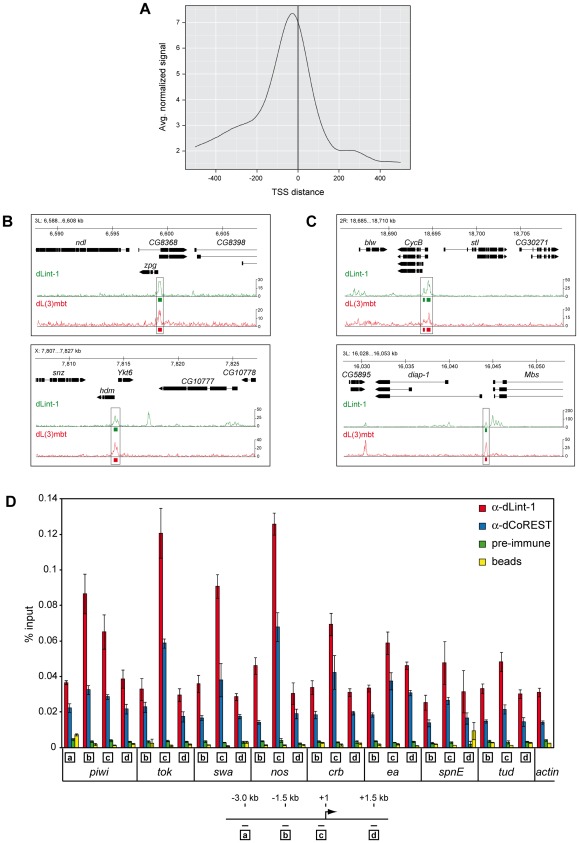
LINT binds to genes deregulated in *l(3)mbt^ts^* brain tumours. (A) Averaged dLint-1 ChIPseq signal intensity around those transcription start sites that are within 1 kilobase of a dLint-1 peak (normalized to 1 million tags). In cases where multiple TSSs were within 1 kb of a peak, the one with the smallest summit-TSS distance was chosen. (B) Genome browser view of dLint-1 and dL(3)mbt [4] chromatin association in regions containing MBTS genes (upper panel: *zpg*, lower panel: *hdm*). (C) Genome browser view of dLint-1 and dL(3)mbt [4] chromatin association in regions containing SWH target genes (upper panel: *CycB*, lower panel: *diap-1*). (D) Chromatin immunoprecipitation (ChIP) of Kc cell chromatin using dLint-1 #1 and dCoREST antibodies. As a control chromatin was precipitated with ProtG beads loaded with pre-immune serum or no antibody (beads). Genes analyzed are denoted below the panel. Amplified regions are indicated by boxed letters and had the following distances from the TSS as illustrated below: a, 3 kb upstream; b, 1.5 kb upstream; c, 0–0.15 kb (promoter); d, 1.5 kb downstream.

We next performed a ChIP-Seq analysis to determine genomewide binding sites of dLint-1 in Kc and S2 cells. dLint-1 binding sites identified in the two cell lines showed a high correlation even though they represent different cell types (Pearson correlation 0.81; Figure S4). This suggests that many dLint-1 binding sites are conserved across cell types. The majority of dLint-1 binding sites in S2 cells map to a 250 bp region surrounding transcriptional start sites (TSSs) (−200 to +50, Figure 4A and Figure S5A). More than half of the dLint-1 peaks overlap with a TSS (Figure S5B). This suggests that dLint-1 is preferentially associated with promoter regions indicating a potential role in transcription regulation.

We included dL(3)mbt ChIP-Seq data from larval brain reported by Richter and colleagues in our analysis to determine the extent of overlap between dL(3)mbt and dLint-1 binding [4]. Of the 2902 dL(3)mbt peaks defined in this study, 2347 (80.1%) overlapped with dLint-1 peaks. Although we are comparing dL(3)mbt binding sites in larval brain tissue with dLint-1 binding sites in S2 cells, these results strongly suggest that dL(3)mbt and dLint-1 bind together to many sites within the genome.

Two recent studies have implicated the derepression of MBTS genes with a germline function and genes targeted by the SWH pathway, respectively, in the formation of brain tumours in *l(3)mbt* mutant larvae [3], [4]. dL(3)mbt associates with many of these genes in larval brain [4]. To determine whether LINT binds these genes as well we compared dL(3)mbt and dLint-1 binding profiles. Figure 4B shows dLint-1 and dL(3)mbt binding profiles at regions containing the MBTS germline genes zero population growth (zpg) and hold'em (hdm). In both cases, dL(3)mbt and dLint-1 cooccupy sequences overlapping with promoter and TSS. Inspection of all 32 MBTS germline genes derepressed in *l(3)mbt* tumours revealed that 25 are bound by dL(3)mbt in larval brain and dLint-1 in both Kc and S2 cells (Table S1). We also assessed dCoREST binding to germline-specific genes by ChIP-qPCR (Figure 4D). Like dLint-1, dCoREST binding peaks at the promoter regions of germline genes. Comparison of the dL(3)mbt and dLint-1 binding profiles in regions containing the 11 SWH target genes analyzed by Richter *et al.* revealed that 6 SWH targets are cooccupied by dL(3)mbt and dLint-1 (Figure 4C and Table S1). Taken together these results suggest that the LINT complex binds to the promoter regions of a majority of MBTS germline and SWH target genes that are deregulated in *l(3)mbt^ts^* tumours.

### LINT represses MBTS genes with germline function

**Figure 5:**
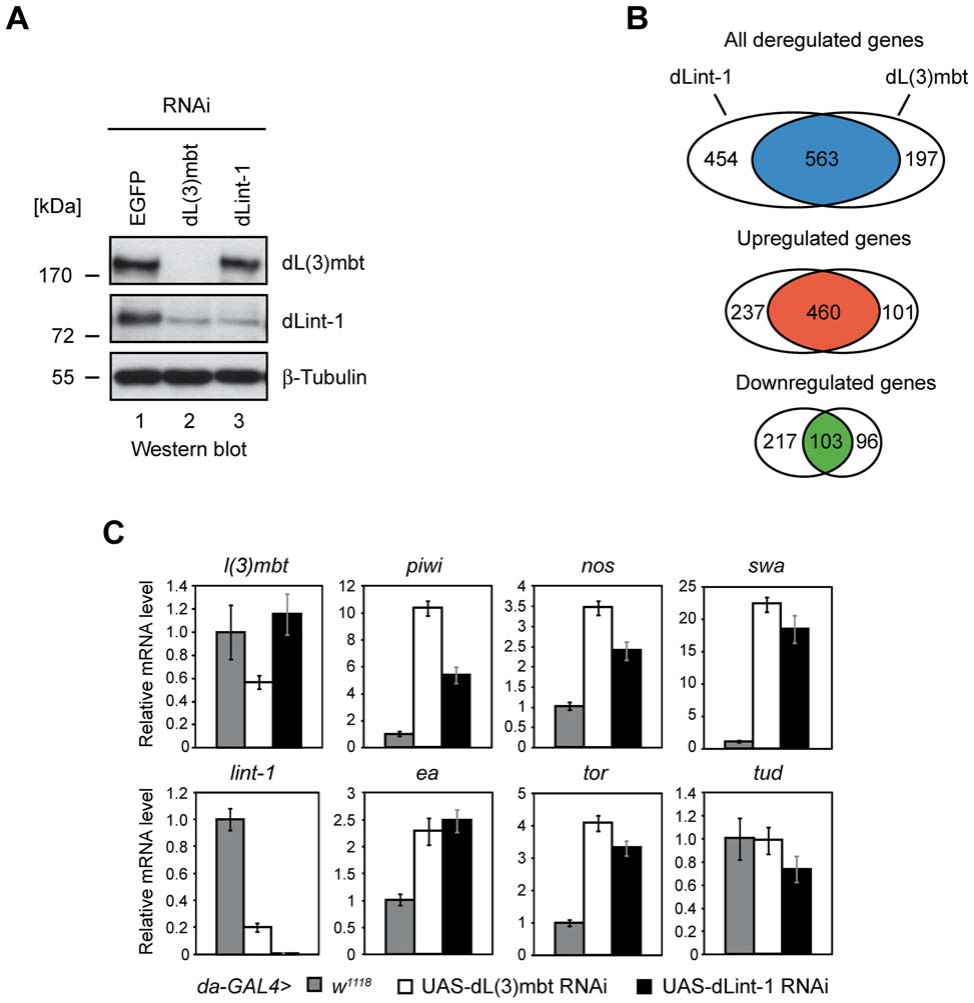
LINT represses MBTS genes with germline function. (A) Gene expression analysis upon RNAi mediated depletion of dL(3)mbt and dLint-1 in Kc cells. Kc cells were treated with double-stranded RNA (dsRNA) directed against EGFP, dL(3)mbt or dLint-1. Nuclear extracts were analyzed by Western blot as indicated. (B) Venn diagrams of dL(3)mbt and dLint-1 regulated genes (fold change ≥1.5, adj. p≤0.05). (C) Target gene expression upon depletion of dL(3)mbt and dLint-1 in flies. RNAi depletion was achieved by crossing the *da-GAL4* driver strain to *w^1118^* (control) and strains carrying dL(3)mbt or dLint-1 RNAi transgenes under UAS control, respectively. RNA was isolated from 3rd instar larvae and transcription was determined by RT-qPCR. Transcription levels in control crosses were set to 1. Tudor serves as a negative control.

To assess if LINT binding to MBTS germline and SWH target genes is functionally relevant we determined changes in the gene expression profile of Kc cells following RNAi-mediated depletion of dL(3)mbt and dLint-1, respectively (Figure 5A). Microarray analysis identified 563 genes that were deregulated by both RNAi treatments (Figure 5B). 460 (81,7%) of these were upregulated suggesting that LINT functions predominantly to repress transcription.

In this experiment none of the 11 SWH target genes was found to be derepressed in dL(3)mbt or dLint-1 depleted Kc cells (Table S2). By contrast, 15 of the 32 MBTS genes with a germline function were upregulated in both dL(3)mbt and dLint-1-depleted cells (Table S2). Moreover, the MBTS germline genes *piwi*, *nos*, *swa*, *hdm*, *RbS5b and CG32313* were among the top 50 genes that were most strongly derepressed in both dL(3)mbt and dLint-1 depleted cells (Table S3). Furthermore, 20 of the top 50 genes encode proteins with a testis- or ovary-specific expression pattern (Table S3).

We sought to determine if RNAi depletion of dL(3)mbt and dLint-1 would also lead to derepression of genes with germline function in developing larvae of transgenic flies. Figure 5C shows that depletion of either LINT subunit resulted in derepression of *piwi*, *nos*, *swa*, *ea* and *tor*. These results suggest that dL(3)mbt and dLint-1 play important roles in the repression of many genes with a germline-specific expression pattern both in cell lines and in the developing fly.

**Figure 6:**
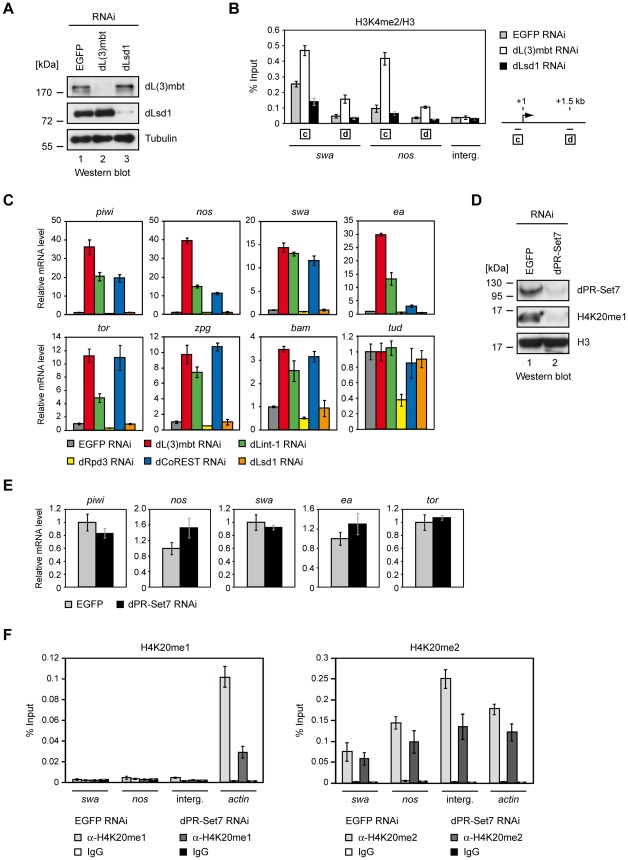
dLsd1, dRpd3, and dPR-Set7 are not essential for MBTS gene repression. (A) Kc cells were treated with dsRNA directed against EGFP, dL(3)mbt and dLsd1. Nuclear extracts of RNAi treated Kc cells were subjected to Western blot and analyzed using antibodies as indicated. (B) Chromatin of RNAi treated cells was precipitated with H3K4me2 and H3 antibodies as indicated. The ratio of H3K4me2 and H3 ChIP signals is shown for *swa* and *nos* promoter and ORF regions and an unrelated intergenic region (interg.). Genes analyzed are denoted below the panel. Amplified regions are indicated by boxed letters and have the following distances from the transcriptional start site as illustrated on the right: c, 0–0.15 kb (promoter); d, 1.5 kb downstream. (C) Kc cells were treated with dsRNA directed against EGFP, dL(3)mbt, dLint-1, dRpd3, dCoREST and dLsd1 as indicated and transcription was determined by RT-qPCR. Transcription levels in EGFP RNAi treated cells were set to 1. Tudor serves as a negative control. (D) Kc cells were treated with dsRNA directed against EGFP and dPR-Set7 as indicated. Nuclear extracts and acid extracted histones were analyzed by Western blot as indicated. (E) Transcription levels of target genes were determined by RT-qPCR. Transcription levels in cells treated with dsRNA against EGFP were set to 1. (F) Chromatin from cells treated with RNAi against dPR-Set7 or EGFP (control) was precipitated with H4K20me1 and IgG (left panel) or H4K20me2 and IgG (right panel) antibodies as indicated. ChIP signals are shown for *swa* and *nos* promoter regions, an intergenic region and the *actin* gene as denoted below the panel.

Given that our biochemical analyses had suggested that dLint-1 and dCoREST in addition to being core components of LINT also associate with the histone modifying enzymes dLsd1 and dRpd3 we considered the role of histone modifications in the repression of MBTS germline genes. We derepressed the MBTS germline genes *swa* and *nos* by RNAi-mediated depletion of dL(3)mbt in Kc cells and determined changes in H3K4 methylation by ChIP (Figure 6A and 6B). Derepression of both genes correlated with increased H3K4me2 ChIP signals in both promoter and coding regions. To test if the H3K4me1/2-specific demethylase dLsd1 might be involved in maintaining low H3K4me2 levels at the repressed *swa* and *nos* genes we depleted dLsd1 by RNAi (Figure 6A). However, this did not result in increased H3K4me2 levels (Figure 6B).

We also tested if depletion of dLsd1 or dRpd3 would be sufficient to derepress LINT target genes (Figure 6C). RNAi-mediated depletion of dL(3)mbt, dLint-1 or dCoREST resulted in strong derepression of 7 out of 8 MBTS germline genes tested. By contrast, depletion of dLsd1 or dRpd3 had no significant effect.

We conclude that derepression of MBTS germline genes can be achieved by depletion of any of the three LINT core components but not by depletion of dLsd1 or dRpd3.

dL(3)mbt binds H4K20me1/2 via its MBT domains *in vitro* [14]. To test if H4K20 methylation contributes to LINT target gene repression we depleted the H4K20-specific methylase dPR-Set7 (Figure 6D). While RNAi treatment resulted in robust depletion of dPR-Set7 and global reduction on H4K20me1 levels, expression of LINT target genes was not affected (Figure 6D and 6E).

We also assessed H4K20me1 and H4K20me2 levels at LINT target genes by ChIP-qPCR (Figure 6F). We used the *actin* gene as a postive control to verify the efficiency of the ChIP. Indeed, robust H4K20me1 levels were detected at the *actin* gene (Figure 6F, left panel). By comparison H4K20me1 levels at the *swa* and *nos* promoters, which bind LINT, were approximately 20-fold lower. H4K20me1 ChIP signals at *swa* and *nos* were comparable to signals at an intergenic region that we used as a negative control. Moreover, H4K20me1 ChIP signals at *swa* and *nos* were not significantly different than signals obtained with the IgG control IP. RNAi-mediated dPR-Set7 depletion resulted in a strong reduction of H4K20me1 levels at the *actin* gene but did not affect H4K20me1-levels at *swa* and *nos*. We confirmed these results using an independent H4K20me1-specific antibody (Figure S6). We conclude that H4K20me1 appears to be absent from the LINT-bound *swa* and *nos* promoters.

H4K20me2 is the most abundant form of histone H4 both in *Drosophila* and mammals accounting for 85–90% of total H4 [10], [11]. Accordingly, ChIP using a H4K20me2-specific antibody produced robust ChIP signals for all four regions tested (Figure 6F, right panel). Depletion of dPR-Set7 reduced H4K20me2 levels at all regions. H4K20me2 levels differed by a factor of less than 3 between *swa*, *nos*, *actin* and the intergenic region. In fact, the LINT-complex bound *swa* and *nos* promoters displayed the lowest H4K20me2 levels of the four. We conclude that H4K20me2 is detectable at LINT target genes. However, presumably due to the abundance of this modification it is uniformely high along the chromosome and also present at control regions that do not bind LINT.

Taken together, these results suggest that the LINT core subunits - but not the histone modifying enzymes dLsd1, dRpd3 and dPR-Set7 - are required to maintain the repression of many germline-specific genes in cell lines and in developing flies. Changes in H3K4 methylation levels detected at the *swa* and *nos* genes are likely to be a consequence of derepression rather than its cause. In addition, our data do not support an important role of H4K20 methylation in the targeting of LINT to promoters.

**Figure 7:**
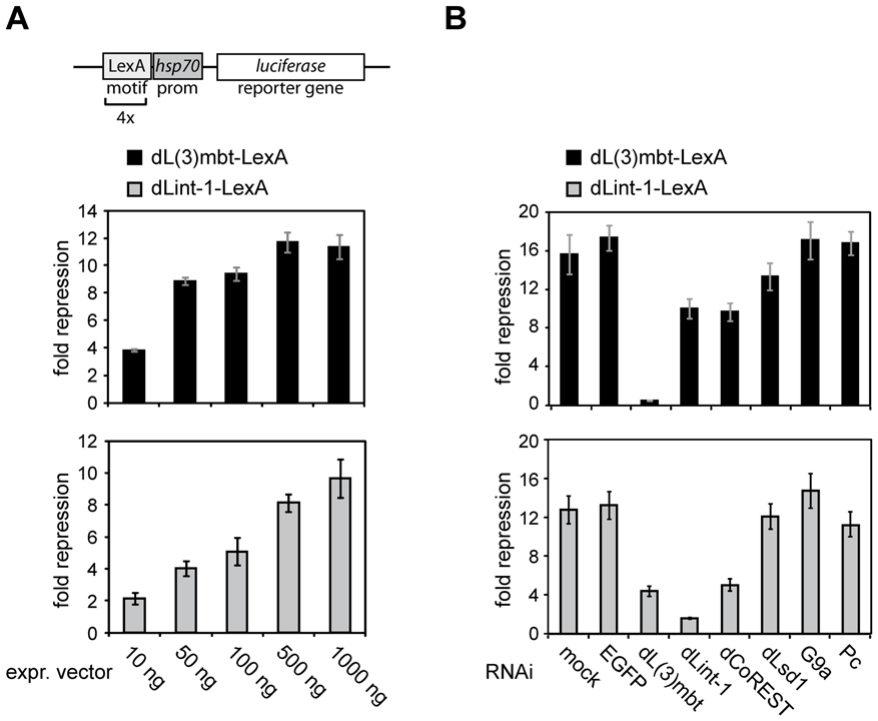
Promoter recruitment of LINT subunits results in transcriptional repression. (A) A *Firefly* luciferase reporter construct (schematic representation on top) was transiently cotransfected into RNAi treated Kc cells along with a *Renilla* luciferase reporter and varying amounts of expression vectors for LexA or dL(3)mbt-/dLint-1-LexA fusion proteins as indicated. Repressor activities of dL(3)mbt-LexA and dLint-1-LexA are presented as -fold repression normalized against activities measured for LexA expression alone. (B) Kc cells were treated with no dsRNA (mock) or dsRNA against EGFP, dL(3)mbt, dLint-1, dCoREST, dLsd1, G9a and Pc. Cells were then cotransfected with reporter and expression vectors as in (A).

Given that LINT-mediated repression appears to be largely independent of histone modifying activities and given that LINT displays a strong preference for binding around the TSS, an alternative repression mechanism could be based on restricting transcription factor and/or RNA polymerase II access to promoter sequences. To test this hypothesis, we recruited LINT subunits to the promoter of a reporter gene (Figure 7A). Indeed, recruitment of a dL(3)mbt- or a dLint-1-LexA fusion protein to a luciferase reporter gene driven by a LexA site containing promoter resulted in robust, dosis-dependent repression. To test the involvement of histone modifications in this system we RNAi depleted subunits of histone modifying complexes with an established role in transcriptional repression (Figure 7B). dLsd1, G9a, Pc, E(z), Suz(12) and dRING did not abrogate repression of the reporter or repression of MBTS germline genes (Figure 7B and *data not shown*). By contrast, depletion of the three LINT subunits dL(3)mbt, dLint-1 and dCoREST resulted in partial derepression suggesting that the presence of all three subunits at the promoter is required for efficient transcriptional repression.

## Discussion

We have identified and characterized LINT a novel dL(3)mbt complex that represses a set of germline-specific genes that is deregulated in malignant tumours of the larval brain. LINT has three core subunits, dL(3)mbt, dLint-1 and dCoREST, all three of which are required to maintain repression of germline-related MBTS genes in cell lines and larvae.

**Figure 8:**
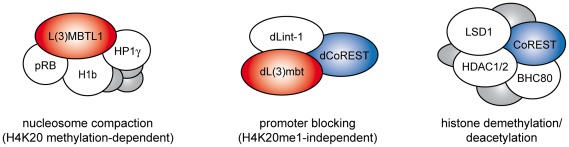
L(3)mbt and LSD1/CoREST complexes. Schematic representation of complex composition of mammalian L3MBTL1 (left), *Drosophila* LINT (middle) and mammalian LSD1/CoREST (right) complexes [5], [21], [22]. Only core subunits are shown. Shared homologous subunits are indicated by color (red: L(3)mbt, blue: CoREST). Proposed repression mechanisms for each complex are indicated below.

LINT subunit composition differs from the human L3MBTL1 complex which contains pRb, HP1γ, H1b and core histones (Figure 8). dLint-1 has no apparent homolog in mammals. The mammalian homologs of dCoREST exist in complexes containing LSD1 and HDAC1/2. dLsd1 and dRpd3 are not stably associated with LINT. Nevertheless, the LINT subunit dLint-1 associates with dCoREST, dLsd1 and dRpd3 arguing for the existence of complexes in *Drosophila* that are related to mammalian CoREST/LSD1 complexes. Two observations are consistent with the view that these complexes might associate with chromatin and occupy sites that are not bound by LINT. First, dLint-1 is associated with approximately 50 bands on polytene chromosomes that show no dL(3)mbt binding. Second, ChIP-Seq analysis has revealed 2,902 dL(3)mbt binding sites but more than 8,000 dLint-1 binding sites. The functional relationship between these different dLint-1-containing complexes is unclear.

Comparison of genomewide binding profiles of dL(3)mbt in larval brain and dLint-1 in S2 and Kc cells strongly argues that LINT subunits bind to a large set of common binding sites. In particular, MBTS germline-related genes are bound and often repressed by the three LINT subunits. Our finding that LINT exists in larval brain strongly implies that it is the LINT complex that is inactivated in *l(3)mbt^ts^* mutants. In addition to MBTS genes, genes targeted by the SWH pathway have recently been shown to be deregulated in *l(3)mbt^ts^* brains [4]. Although we have detected binding of dLint-1 to about half of the SWH targets, we have not detected changes in SWH target gene expression following depletion of dL(3)mbt or dLint-1 in Kc cells. It is possible that protein depletion was not sufficient to derepress these genes under our conditions. Also, SWH target genes might be regulated differently in larval brain compared to cell lines.

Our results suggest that maintenance of MBTS germline gene repression by LINT is largely independent of repressive histone modifying activities. Depletion of the dLint-1-associated dLsd1 and dRpd3 enzymes does not lead to derepression of LINT targets. We detected an increase of the active H3K4me2 mark at derepressed LINT target genes but this is most likely a result of active transcription rather than a direct consequence of the loss of LINT associated chromatin modifying activities. In agreement with this view, depletion of dLsd1 does not result in changes of H3K4me2 levels at LINT target genes. Our microarray analysis also did not detect significant changes in the expression of genes recently shown to be repressed by dLsd1 in S2 cells and developing flies [12], [13]. This suggests that LINT and dLsd1 target different sets of genes.

Chromatin association and the repressive potential of human L3MBTL1 is enhanced by PR-SET7 and H4K20 monomethylation [5], [6]. Depletion of dPR-Set7, the sole *Drosophila* enzyme responsible for H4K20 monomethylation, did not result in derepression of LINT targets. We also did not detect significant levels of H4K20me1 at promoters of LINT target genes. This strongly suggests that even though dL(3)mbt can bind H4K20me1 *in vitro* this interaction does not play an important role in LINT complex targeting and repression.

dL(3)mbt does also bind to H4K20me2 *in vitro*. Indeed, H4K20me2 is present at LINT-regulated genes. However, H4K20me2 levels are are not elevated at LINT target gene promoters compared to control regions. This finding was not surprising given that 85–90% of all histone H4 molecules are dimethylated at K20 and, therefore, H4K20me2 levels might be expected to be uniformely high along the chromosome [10], [11]. This makes it unlikely that an interaction between the MBT domains and H4K20me2 specifically directs the LINT complex to its target genes. However, it remains possible that after recruitment of LINT by other means, an interaction between dL(3)mbt and H4K20me2 contributes to transcriptional repression.

Depletion of other enzymes setting repressive histone marks such as H3K9me3 and H3K27me3 has likewise no effect on LINT-mediated repression. Although we have not been able to test all histone modifying enzymes for their roles in LINT target gene repression, the results argue for a largely histone modification independent mode of repression. LINT subunits bind predominantly near TSSs suggesting that LINT might inhibit transcription by restricting the access of RNA polymerase II or transcription factors to promoters. In support of this model, recruitment of LINT subunits to the promoter of a reporter gene is sufficient for repression even under conditions where the levels of repressive histone modification enzymes are reduced. We can envisage two modes of promoter access restriction by LINT that are not mutually exclusive. First, LINT might bind to the promoter segments required for RNA polymerase II recruitment. Second, as has been suggested for human L3MBTL1, LINT might locally compact nucleosomes. Two of our findings are inconsistent with the latter hypothesis. Nucleosome compaction by L3MBTL1 is dependent on the presence of the H4K20me1 modification. However, as discussed above, ablation of this modification does not result in derepression of LINT target genes. In addition, as a consequence of nucleosome compaction at LINT bound promoters one might expect a local increase in nucleosome density. However, histone H3 ChIP experiments have shown that the promoters of LINT target genes are generally depleted of nucleosomes (*data not shown*). While these findings do not rule out a local nucleosome compaction that is - once established - independent of H4K20 monomethylation and undetectable by H3 ChIP, we favour the simpler hypothesis that LINT association with promoter sequences prevents transcription factors and RNA polymerase II from promoter binding (Figure 8).

The dL(3)mbt and dCoREST subunits of LINT are well conserved. Similar to the derepression of germline-related genes in *l(3)mbt^ts^* tumours, misexpression of testis-specific genes (so-called cancer testis antigens) have been described in many human tumours [14]. Based on our study, it is conceivable that L3MBTL1 or CoREST play a role in the repression of cancer testis antigens.

## Materials and Methods

### Cell culture, transfections, RNAi treatments, and baculovirus infection

*D. melanogaster* and *S. frugiperda* cell lines were maintained under standard conditions. Transfection of Kc cells for the luciferase reporter assay was carried out according to the manufacturer's instructions (Qiagen). RNA interference and baculovirus infection were described in [15].

### Antibodies

Rabbit polyclonal and rat monoclonal (MBT P1 6E6) anti-dL(3)mbt, rabbit polyclonal anti-dMi-2 (anti-dMi2-Nterm) and anti-dRpd3 antibodies have been previously described [16], [17]. Rabbit dL(3)mbt antibody was used in Western blot and monoclonal dL(3)mbt antibody was used for immunostainings. dCoREST (G. Mandel), dSu(var)3-3 (dLsd1, T. Rudolph), dPR-Set7 (A. Imhof) and RBF2 (N. Dyson) antibodies were generous gifts. anti-beta-Tubulin (clone KMX-1) antibody was from Millipore, anti-FLAG M2 antibody and agarose from Sigma. Histone antibodies were purchased from the following companies: Abcam: anti-H4K20me1 (#9051); anti-H3 (#1791); Millipore: anti-H4K20me1 (#17-651); anti-H3K4me2 (clone CMA303, #05-1338); Active Motif: anti-H4K20me2 (#39173). Anti-dLint-1 antibodies were developed in this study (Figure 2A). Anti-dLint-1 #1 antibody was used for immunoprecipitation, immunostaining and ChIP analysis. Anti-dLint-1 #2 antibody was used for immunoblotting.

To generate Lint-1-specific antibodies, the C-terminus (aa 302–602) of dLint-1 was fused to GST by cloning into pGex4T1 vector. The recombinant protein was expressed in *E. coli* BL21 according to standard procedures. The GST-Lint-1-C-term fusion protein was purified via affinity chromatography using a GSTrap FF column (GE Healthcare) and subsequent ion exchange chromatography using a HiTrap SP HP column (GE Healthcare) on an Äkta purifier system (GE Healthcare) according to the manufacturer's instructions. For immunization two rabbits (serum #1 and #2) were injected with 0.5 mg of purified GST-Lint-1-C-term fusion protein each (Peptide Speciality Laboratories, Heidelberg, Germany). The specificity of antibodies was verified by RNA interference in Kc cells and subsequent Western blot analysis of nuclear extracts (Figure 2A).

### Immunostaining and immunoblotting

The following antibodies were used: Primary antibodies: anti-dL(3)mbt P1 6E6 (rat, concentrated) 1∶2 and dLint-1 #1 (rabbit) 1∶50. Secondary antibodies: Alexa Fluor 546 goat anti-rat and Alexa Fluor 488 goat anti-rabbit 1∶200 (Invitrogen). Analysis was performed with a Zeiss fluorescence microscope (Axioplan). Quantitative analysis of dL(3)mbt and dLint-1 binding sites was accomplished by visual inspection using Image J software (http://rsbweb.nih.gov/ij/). Western blots were carried out as previously described [16].

### Transgenic fly lines and polytene chromosomes

Transgenic fly lines were generated and polytene chromosomes were analyzed by immunofluorescence as described in [18]. RNA interference experiments in flies were performed using stocks from the VDCR phiC31 RNAi Library (http://stockcenter.vdrc.at/control/main) carrying RNAi transgenes under UAS control (transformant IDs: dL(3)mbt: 104563; dLint-1: 105932). For overexpression or knockdown experiments the *GAL4*-driver strains *da*-*GAL4* and *sgs58AB*-*GAL4* were used, respectively. As control, *GAL4*-driver strains were crossed with the host strain *w^1118^*.

### Expression of recombinant proteins

dL(3)mbt and dLint-1 cDNA was obtained from BDGP (clone LD05287 of dL(3)mbt and RE35228 of dLint-1). Vectors for expression of N-terminally FLAG-tagged dL(3)mbt and C-terminally FLAG-tagged dLint-1 in S2 cells were generated by PCR-cloning of the respective ORFs in either pPac-HA-FLAG or pPac-FLAG-Back vector using gene specific primers. Generation of S2 cell lines stably expressing recombinant dL(3)mbt and dLint-1 was performed as described previously [17]. To obtain recombinant dL(3)mbt-FLAG and dLint-1 baculovirus transfer vectors ORFs were cloned into pVL1392 using appropriate sets of primers. Baculovirus production and purification have been previously described [16].

### Interaction assay of in vitro translated ^35^S labeled dLint-1 and baculoviral expressed FLAG–dL(3)mbt

The dLint-1 coding region was inserted into pING14A. *In vitro* translation of dLint-1 and a luciferase control in the presence of ^35^S-methionine was carried out with the TNT SP6 Coupled Reticulocyte Lysate System (Promega) according to the manufacturer's instructions. FLAG-tagged dL(3)mbt was expressed in Sf9 cells using the baculovirus system. 12 ml of Sf9 extracts, were bound to 60 µl of anti-FLAG M2 agarose (Sigma) and washed extensively with LyBu200 and LyBu500 buffer (20 mM Hepes pH 7.6, 200 mM or 500 mM KCl, 10% glycerol, 0.1% NP-40, 1 mM DTT and protease inhibitors). 10 µl of anti-FLAG beads were blocked with 0.2 µg/µl BSA for 30 min and incubated with 8 µl of ^35^S-dLint-1 or 6 µl of ^35^S-Luciferase for 3 hours each in IP buffer (25 mM Hepes, pH 7.6, 12.5 mM MgCl_2_, 0.1 mM EDTA, pH 8.0, 10% glycerol, 0.1% NP40, 150 mM NaCl) at 4°C. After extensive washing with IP buffer samples were separated by SDS-PAGE and treated with Amplify (Amersham) according to manufacturer's instructions. The gel was dried and subjected to autoradiography.

### Extract preparation, histone extraction, and immunoprecipitations

Nuclear extract from Kc and S2 cells and whole cell extracts from Sf9 cells were prepared and immunoprecipitations were carried out as described elsewhere [16]. For extraction of histones the insoluble nuclear pellet was resuspended in 0.4 M HCl and incubated overnight at 4°C with shaking. After centrifugation (at 4°C, 15 min, 13000 rpm) extracted histones were in the supernatant. For larval brain extracts, brains were resuspended in LNBI buffer (15 mM Hepes/KOH, pH 7.6, 10 mM KCl, 5 mM MgCl_2_, 0.1 mM EDTA, pH 8.0, 350 mM sucrose, 1 mM DTT, protease inhibitors), homogenized and incubated for 10 min on ice. The tissue suspension was then centrifuged, nuclei were resuspended in LNBII buffer (15 mM Hepes/KOH, pH 7.6, 385 mM KCl, 5 mM MgCl_2_, 0.1 mM EDTA, pH 8.0, 0.1% Tween 20, 10% glycerol, 1 mM DTT, protease inhibitors), incubated for 30 min on ice and centrifuged. LNBI and LNBII extracts were pooled and used for immunoprecipitation. For immunoprecipitation 750 µg of nuclear extract (from Kc cells or 0–12 h embryos) or protein extract from 200 brains were incubated with 1.5 µl dLint-1 #1 antibody diluted with IP buffer or PBS, respectively, to a final salt concentration of 200 mM, and incubated for 2 h to 3 h at 4°C with rotation. 5 µl of Protein G beads (GE Healthcare) were added and incubation was continued for 1 h. Following extensive washing with IP buffer or PBS, immunoprecipitates were analyzed by Western blot. FLAG-immunoprecipitations were carried out with S2 nuclear extracts (750 µg total protein) and 5 µl anti-FLAG M2 agarose (Sigma), diluted with IP buffer to a final salt concentration of 200 mM. Incubation was performed for 3 h at 4°C with rotation. After extensive washing with IP buffer beads were analyzed by Western blotting. 200 µl of whole cell extracts of baculovirus-infected Sf9 cells were incubated in 800 µl IP buffer with 10 µl anti-FLAG M2 agarose for 3 h at 4°C with rotation. Beads were washed extensively with IP buffer and analyzed by Western blotting.

### Complex purification

For FLAG affinity purification of FLAG-dL(3)mbt and dLint-1-FLAG associated proteins, nuclear extracts from stable S2 cell lines were prepared, as described before. 70 mg (total protein) of FLAG-dL(3)mbt extract and 150 mg (total protein) of dLint-1-FLAG extract, as well as an equal amount of S2 mock extract were incubated with 60 or 120 µl FLAG M2 agarose (Sigma-Aldrich; equilibrated in D-125/10) respectively in D-125 (D-x: 20 mM HEPES/KOH pH 7.6, x mM KCl, 2 mM MgCl_2_, 0.2 mM EDTA, 0.05% NP40, 10% glycerol, protease inhibitors) buffer at 4°C for 3 hr with rotation. FLAG-beads were washed once with buffer D-125, three times with D-300 and once with D-125. Each wash was carried out with 10 ml of buffer at 4°C for 10 min on a rotating wheel. Bound proteins were eluted with 0.4 mg/ml FLAG-peptide (Sigma-Aldrich) in D-125 buffer. The beads were diluted 1∶1 in elution buffer and elution was carried out for 2 hr on ice with regular mixing of the slurry. Additionally, an elution was performed overnight at 4°C on a shaker. Eluates were combined, precipitated using StrataClean resin (Stratagene), subjected to SDS-PAGE and visualized by silver or Colloidal Coomassie Blue (Invitrogen) staining. In general, 10% of the eluates were visualized by silver staining, whereas 90% were loaded onto a gel for Colloidal Coomassie Blue staining. Protein bands were excised from Colloidal Coomassie Blue stained gels and analyzed by peptide mass fingerprinting (Zentrum für Proteinanalytik, Munich, Germany).

Ion exchange chromatography (IEX) was carried out according to standard procedures on an Äkta purifier system with a Frac-950 fraction collector using columns supplied by GE Healthcare according to the manufacturer's instructions. Kc nuclear extract was diluted 4.2× with IEX-0 buffer (20 mM Tris/HCl pH 7.5, x mM NaCl, 1 mM DTT, PMSF) to adjust the NaCl concentration of the sample to 100 mM. Subsequently, the sample was bound to a HiTrap Q Sepharose FF column (1 ml volume), that was prior to this equilibrated with IEX-100 buffer. The flow through was loaded once more to ensure efficient binding of proteins. Then the column was washed with 5–10 ml of IEX-100 buffer or until no protein (measured by absorption at 280 nm) appeared in the effluent. Elution was performed in two steps: First, applying IEX-500 buffer and second, applying IEX-1000 buffer. Peak fractions of the eluates were collected and tested together with the flow through by Western blotting. Next, the eluate (IEX-500 peak), containing the proteins of interest, was diluted 5× with IEX-0 buffer. The sample was then bound to a Mono Q 5/50 GL column (GE Healthcare) and after the application the column was washed with 5–10 ml of IEX-100 buffer or until no protein was present in the effluent. For elution a continuous salt gradient was used, going from 100 mM up to 500 mM NaCl in IEX buffer, applied at a flow rate of 1 ml/min, collecting 50 fractions each of 0.5 ml volume. Finally, residual protein was eluted in one step with IEX-1000 buffer. 500 µl fractions were collected, precipitated using StrataClean resin (Stratagene) and subjected to Western blot analysis.

### Luciferase assay

Kc cells were treated with dsRNA for 48 hours. Then cells were transfected with the following expression vectors using Attractene tranfection reagent (Qiagen): 1. 1 µg of pAc5.1-LexA, pAc5.1-dL(3)mbt-LexA or pAc5.1-dLint-1-LexA; 2. 250 ng of pGL2-hse/lexA; 3. 100 ng of pPacRNLuc. 48 hours after transfection, Firefly and Renilla luciferase activities were measured using the Dual-Luciferase Reporter Assay System (Promega) according to manufacturer's instructions. For each sample the mean from triplicate measurements Firefly and Renilla Luciferase activity and standard deviations were determined. Firefly Luciferase activity was normalized against Renilla Luciferase activity to control for variation in transfection efficiency. Repressive activity (fold repression) was determined by relating luciferase values obtained after expression of LexA fusion proteins to luciferase values obtained after expression of LexA alone.

### qRT–PCR

Total RNA from Kc cells or 3rd instar larvae was isolated using the peqGOLD total RNA kit (Peqlab). 1.5 µg of RNA was applied to RT by incubation with 0.5 µg of Oligo(T)17 primer and 100 U of M-MLV reverse transcriptase (Invitrogen). cDNA was analyzed by qPCR, which was performed using Absolute SybrGreen Mix (Thermo Fisher) and the Mx3000P real-time detection system (Agilent). All amplifications were performed in triplicates using 0.6 µl of cDNA per reaction. Triplicate mean values were calculated according to the ΔΔCT quantification method using GAPDH1 transcription as normalisation reference. Standard deviation was calculated from triplicates, error bars are indicated accordingly. Relative mRNA levels in EGFP RNAi treated Kc cells or control flies were set to 1 and other values were expressed relative to this.

### ChIP

ChIPs were performed as described in the Upstate Biotechnology ChIP assay protocol. 100*10^6^ Kc cells were fixed in 1% formaldehyde for 10 min at RT. Fixation was stopped by the addition of 240 mM glycine. Cells were harvested, washed in ice cold PBS, resuspended in 1 ml SDS-Lysis buffer (50 mM Tris-HCl, pH 8.0, 10 mM EDTA, 1% SDS, protease inhibitors) and incubated for 10 min on ice. Lysates were sonicated using a Bioruptor (Diagenode) to obtain an average fragment length of 0.5 kb and centrifuged (at 4°C, 15 min, 13000 rpm). Shearing of the DNA was analyzed by agarose gel electrophoresis following reversal of crosslinks. The supernatant (chromatin) was subjected to ChIP analysis. 140 µl of chromatin were used per ChIP, diluted 10× with IP-buffer (16.7 mM Tris-HCl, pH 8.0, 16.7 mM NaCl, 1.2 mM EDTA, 0.01% SDS, 1.1% Triton X-100, protease inhibitors) and pre-cleared with 40 µl of pre-blocked ProtG beads (1 mg/ml BSA, 4 h) (GE Healthcare) for 30 min with rotation. The following amounts of antibodies were used for ChIP: anti-dLint-1 #1 (8 µl/ChIP), anti-dCoREST (3 µl/ChIP), anti-H3 (1 µl/ChIP), anti-H3K4me2 (3 µl/ChIP), anti-H4K20me1 (Abcam, 10 µl/ChIP), anti-H4K20me1 (Millipore, 0.25 µl/ChIP), anti-H4K20me2 (10 µl/ChIP). As controls ChIPs were performed ommiting antibody (beads control) or with pre-immune serum. Incubation with antibodies was performed overnight, prior to incubation for 2 h with 35 µl of 1∶1 ProtG slurry at 4°C. Precipitates were serially washed for 10 min, three times with low salt wash buffer (20 mM Tris-HCl pH 8.0, 150 mM NaCl, 2 mM EDTA, 0.1% SDS, 1% Triton-X-100), three times with high salt wash buffer (20 mM Tris-HCl pH 8.0, 500 mM NaCl, 2 mM EDTA, 0.1% SDS, 1% Triton-X-100), once with LiCl wash buffer (10 mM Tris pH 8.0, 250 mM LiCl, 1 mM EDTA, 1% NP-40, 1% sodium deoxycholate), once with TE buffer. Immunoprecipitates were eluted twice with 250 µl elution buffer (1% SDS, 0.1 M NaHCO_3_) for each 15 min at RT and crosslinks were reversed by addition of 20 µl 5 M NaCl and heating at 65°C overnight. Following addition of 10 µl of 0.5 M EDTA, 20 µl 1 M Tris-HCl, pH 6.5 and 2 µl of 10 mg/ml Proteinase K samples were incubated for 1 h at 45°C. DNA was purified with peqGOLD Cycle-Pure Kit (Peqlab) and subjected to gene-specific qPCR. Amplifications were performed in triplicates and mean values were expressed as percentage of input compared with pre-immune serum control. Standard deviation was calculated from the triplicates, and error bars are indicated accordingly.

### ChIP–Seq

ChIP–Seq was carried out on an Illumina Genome Analyzer IIx according to the manufacturer's instructions. Raw Illumina sequence reads (36 bp) were approximately counted using a bloom filter (collision probability 10∧−8) and aligned to the *Drosophila melanogaster* genome (Ensembl 63) with bowtie 0.12.7 [19] allowing at most two mismatches (-n 2) with a mismatch quality sum of 70 (-e 70) and restricting to exactly one mapped location (-m 1 -k 1). Peak calling was performed with MACS [20] (1.4.0rc2 20110214 (Valentine), modified to read BAM files enhanced with a count for each read). MACS allowed a maximum of 3 repetitions of each (position, strand) tuple to exclude PCR artifacts, after a poisson distribution based argument on the repetition probability. The same de-deduplication was applied through out our analysis. For comparison of different lanes, read counts were normalized to 1 million uniquely mapping reads. Peaks from different conditions were considered overlapping when they shared at least 1 bp. Gene annotation was obtained from Ensembl revision 63. Transcription start sites were extracted from Ensembl transcript annotations to include internal TSSs.

### Microarray analysis

Expression analysis was performed using Affymetrix Gene 2.0 microarrays following standard protocols. RNAi to deplete dL(3)mbt and dLint-1 was performed as described above in three biological replicates. RNAi directed against EGFP was performed as a control. Total RNA was extracted from RNAi-transfected cells after 5 d using the peqGOLD total RNA kit (Peqlab). Samples were prepared according to standard Affymetrix protocols using the GeneChipFluidics Station 450 (protocol FS450_0002) and hybridized to the Affymetrix GeneChip Drosophila Genome 2.0 Array. Scans were carried out on an Affymetrix GeneChip Scanner GSC3000_7G and the fluorescence intensities were analyzed with Affymetrix GCOS Software 1.4. Raw data were normalized with the gcrma package of Bioconductor (http://www.bioconductor.org). Gene lists were filtered with the following threshholds: fold change ≥1.5, adj. p≤0.05 (Benjamin Hochberg correction).

### Primers

Primers used for RT–qPCR, ChIP–qPCR, and cloning experiments are listed in Table S4.

## Supporting Information

Figure S1 Purification and identification of dL(3)mbt and dLint-1 interacting proteins. (A) Stable expression of FLAG-dL(3)mbt in S2 cells. Nuclear extracts from control cells (mock, lanes 1 and 2) and cells stably expressing FLAG-dL(3)mbt (lanes 3 and 4) were immunoprecipitated with FLAG antibody (lanes 2 and 4) and analyzed by Western blot as indicated. Lanes 1 and 3: 5% input. (B) FLAG affinity purification of FLAG-dL(3)mbt stably expressed in S2 cells (left panel, compare Figure 1B). Bands that were excised and analyzed by peptide mass fingerprinting (right panel) are denoted on the right with capital letters. (C) Stable expression of dLint-1-FLAG in S2 cells. Nuclear extracts from control cells (mock, lanes 1 and 2) and two S2 cell lines stably expressing dLint-1-FLAG (line #1: lanes 3 and 4; line #2: lanes 5 and 6) were precipitated with FLAG antibody. Immunoprecipitates were analyzed by Western blot using antibodies as indicated (lanes 2, 4 and 6). Lanes 1, 3 and 5: 5% input. (D) FLAG affinity purification of dLint-1-FLAG stably expressed in S2 (line #2) cells (left panel, compare Figure 1E). Bands that were excised and analyzed by peptide mass fingerprinting (right panel) are denoted on the right with capital letters. Note that dCoREST and dLsd1 comigrate and were identified from the same band. (B) and (D) Mass spectrometry data are expressed as probability based molecular weight search (Mowse) scores, including the number of peptides, which matched the identified protein (queries matched). Scores, greater than 60, are significant (p<0.05). Identified polypeptides are given with the according GI number in NCBI, the protein name, if available and the CG gene number, including the corresponding isoform.
(TIF)

Figure S2 Alignment of PHD-like motifs of dLint-1 *Drosophila* homologues. Multiple sequence alignment of dLint-1 (CG1908) *Drosophila* homologues, generated with ClustalW2 program. *Drosophila* species are denoted on the left. The C4HC3 PHD-like motif is written in bold and depicted below the alignment. Cys and His residues are colour-coded in yellow and green. Basic residues (Arg and Lys) are illustrated in red and acidic residues (Asp and Glu) in blue. Positions of amino acid residues (referring to the full length protein) of *D. melanogaster* and other *D.* species, are depicted on top and on the right, respectively. Conservation of residues is displayed below the multiple alignment as follows: ‘*’: Identical residues; ‘:’: conserved substitutions; ‘.’: semi-conserved substitutions.
(TIF)

Figure S3 dLint1 interacting proteins coimmunoprecipitate from embryo extracts. Nuclear extracts from 0 to 12 hr old *Drosophila* embryos were precipitated with protein G beads (beads control, lane 3) and beads loaded with dLint-1 #1 antibody (lane 2) and analyzed by Western blot as indicated (lanes 2 and 3). dMi-2 served as a negative control. Lane 1: 5% input; lane 4 contains dLint-1 antibody (antibody control).
(TIF)

Figure S4 Comparison of dLint1 ChIP-Seq peaks obtained from two different cell lines. Peaks identified in either S2 or Kc ChIP-Seq data were merged. For each resulting region (possibly spawning multiple peaks) tag count normalized to one million reads was log 2 transformed and plotted. Color indicates whether a region was called by MACS in S2 (green), Kc (blue) or both conditions (red).
(TIF)

Figure S5 dLint-1 peaks cluster around TSSs. (A) Approximately 58% of dLint-1 peaks identified in S2 cells overlap with a known transcription start site (TSS). (B) Histogram depicting the distribution of distances from dLint1 peak summits (i.e. region of highest signal intensity) to the next TSS. Distances above 1000 bp were truncated to 1000 bp.
(TIF)

Figure S6 LINT target promoters are devoid of H4K20 mono-methylation. Chromatin from cells treated with RNAi against dPR-Set7 or EGFP (control) was precipitated with H4K20me1 or IgG antibodies as indicated. ChIP signals are shown for *swa* and *nos* promoter regions, an intergenic region and the *actin* gene as denoted below the panel.
(TIF)

Table S1 dL(3)mbt and dLint-1 bind to germline-specific MBTS and SWH target genes. Genes were visually inspected for dL(3)mbt peaks (Richter et al. 2011), dLint-1 peaks in Kc cells and dLint-1 peaks in S2 cells. +: at least one peak.
(DOC)

Table S2 dL(3)mbt and dLint-1 regulate germline-specific MBTS genes. Genes with a fold change ≥1.5 (adj. p≤0.05) were considered deregulated.
(DOC)

Table S3 Top 50 genes repressed by Lint-1.
(DOC)

Table S4 Primer sequences.
(DOC)
