# Supplementary figures and images for: LINT, a Novel dL(3)mbt-Containing Complex, Represses Malignant Brain Tumour Signature Genes
Source: PLoS Genet. 2012 May 3;8(5):e1002676. doi: 10.1371/journal.pgen.1002676 (PMC3342951; doi:10.1371/journal.pgen.1002676)

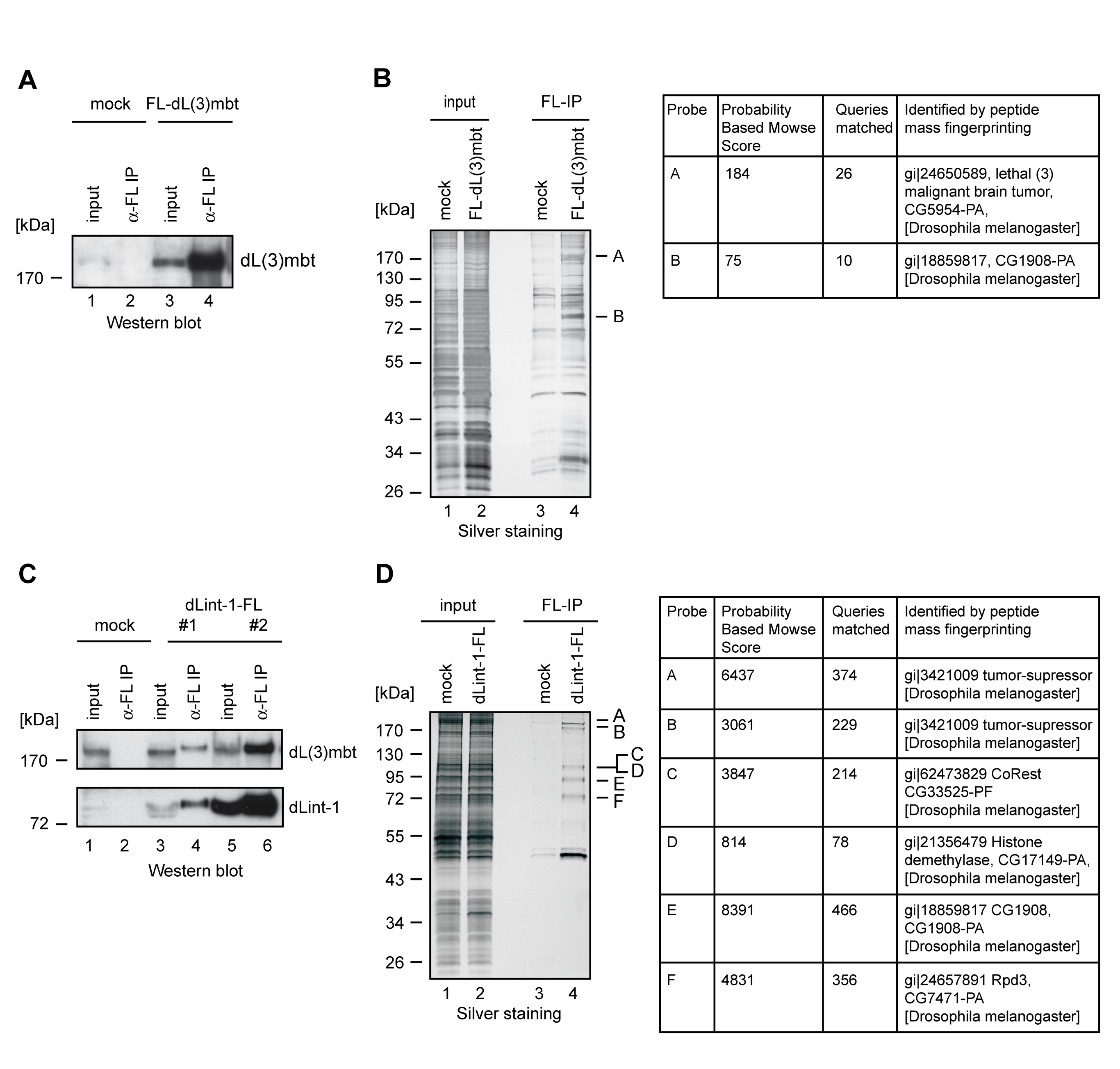

Supplement: Figure S1 — Purification and identification of dL(3)mbt and dLint-1 interacting proteins. (A) Stable expression of FLAG-dL(3)mbt in S2 cells. Nuclear extracts from control cells (mock, lanes 1 and 2) and cells stably expressing FLAG-dL(3)mbt (lanes 3 and 4) were immunoprecipitated with FLAG antibody (lanes 2 and 4) and analyzed by Western blot as indicated. Lanes 1 and 3: 5% input. (B) FLAG affinity purification of FLAG-dL(3)mbt stably expressed in S2 cells (left panel, compare Figure 1B). Bands that were excised and analyzed by peptide mass fingerprinting (right panel) are denoted on the right with capital letters. (C) Stable expression of dLint-1-FLAG in S2 cells. Nuclear extracts from control cells (mock, lanes 1 and 2) and two S2 cell lines stably expressing dLint-1-FLAG (line #1: lanes 3 and 4; line #2: lanes 5 and 6) were precipitated with FLAG antibody. Immunoprecipitates were analyzed by Western blot using antibodies as indicated (lanes 2, 4 and 6). Lanes 1, 3 and 5: 5% input. (D) FLAG affinity purification of dLint-1-FLAG stably expressed in S2 (line #2) cells (left panel, compare Figure 1E). Bands that were excised and analyzed by peptide mass fingerprinting (right panel) are denoted on the right with capital letters. Note that dCoREST and dLsd1 comigrate and were identified from the same band. (B) and (D) Mass spectrometry data are expressed as probability based molecular weight search (Mowse) scores, including the number of peptides, which matched the identified protein (queries matched). Scores, greater than 60, are significant (p<0.05). Identified polypeptides are given with the according GI number in NCBI, the protein name, if available and the CG gene number, including the corresponding isoform. (TIF) [file pgen.1002676.s001.tif]

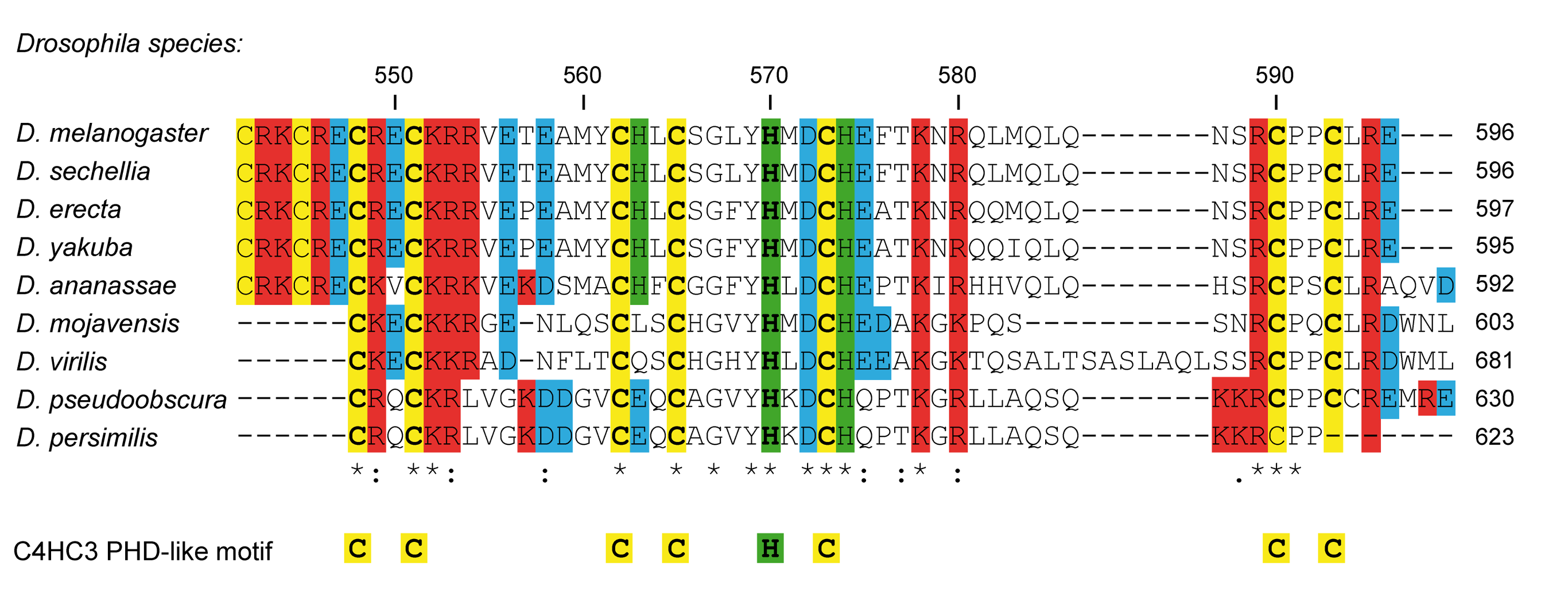

Supplement: Figure S2 — Alignment of PHD-like motifs of dLint-1 Drosophila homologues. Multiple sequence alignment of dLint-1 (CG1908) Drosophila homologues, generated with ClustalW2 program. Drosophila species are denoted on the left. The C4HC3 PHD-like motif is written in bold and depicted below the alignment. Cys and His residues are colour-coded in yellow and green. Basic residues (Arg and Lys) are illustrated in red and acidic residues (Asp and Glu) in blue. Positions of amino acid residues (referring to the full length protein) of D. melanogaster and other D. species, are depicted on top and on the right, respectively. Conservation of residues is displayed below the multiple alignment as follows: ‘*’: Identical residues; ‘:’: conserved substitutions; ‘.’: semi-conserved substitutions. (TIF) [file pgen.1002676.s002.tif]

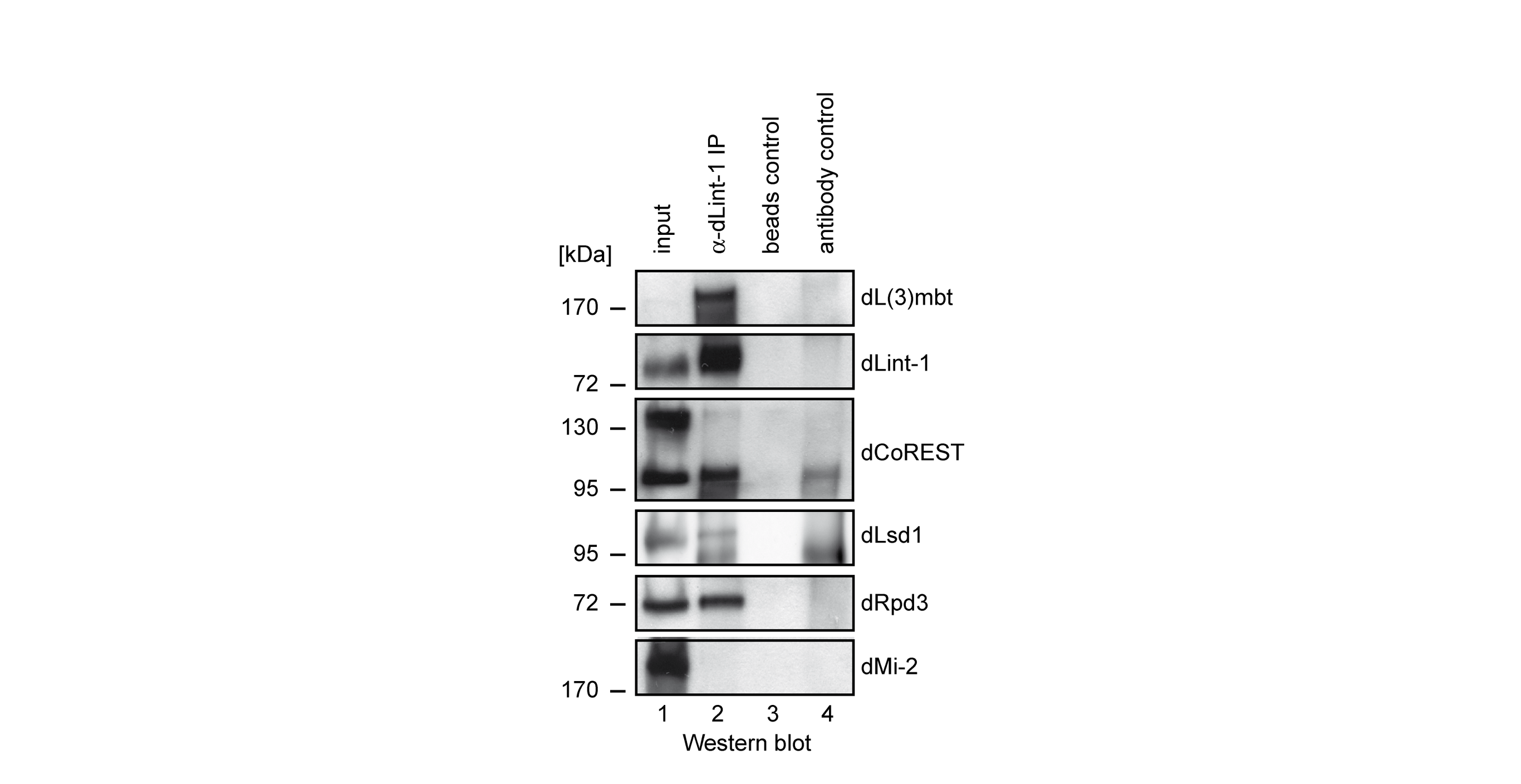

Supplement: Figure S3 — dLint1 interacting proteins coimmunoprecipitate from embryo extracts. Nuclear extracts from 0 to 12 hr old Drosophila embryos were precipitated with protein G beads (beads control, lane 3) and beads loaded with dLint-1 #1 antibody (lane 2) and analyzed by Western blot as indicated (lanes 2 and 3). dMi-2 served as a negative control. Lane 1: 5% input; lane 4 contains dLint-1 antibody (antibody control). (TIF) [file pgen.1002676.s003.tif]

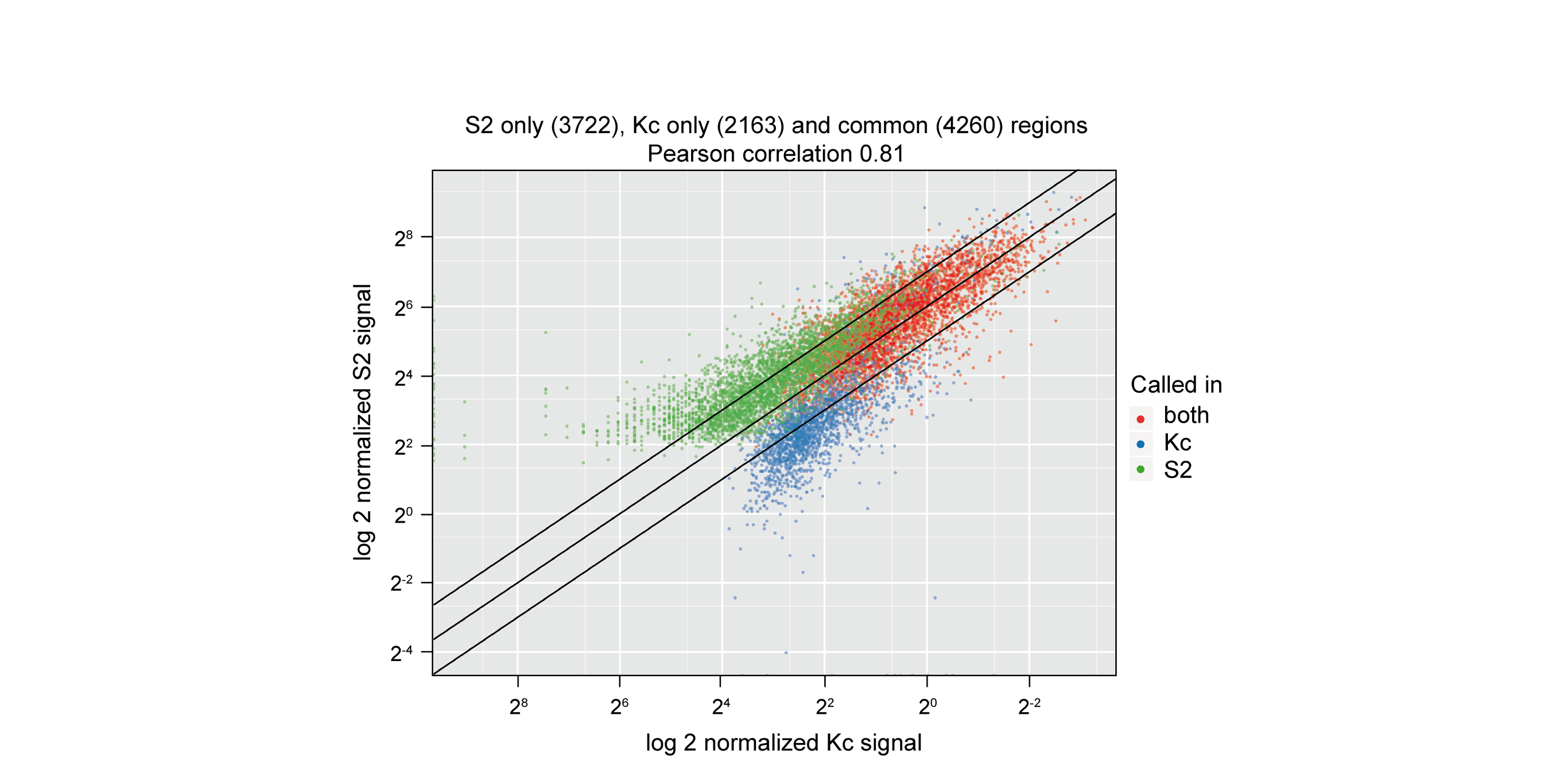

Supplement: Figure S4 — Comparison of dLint1 ChIP-Seq peaks obtained from two different cell lines. Peaks identified in either S2 or Kc ChIP-Seq data were merged. For each resulting region (possibly spawning multiple peaks) tag count normalized to one million reads was log 2 transformed and plotted. Color indicates whether a region was called by MACS in S2 (green), Kc (blue) or both conditions (red). (TIF) [file pgen.1002676.s004.tif]

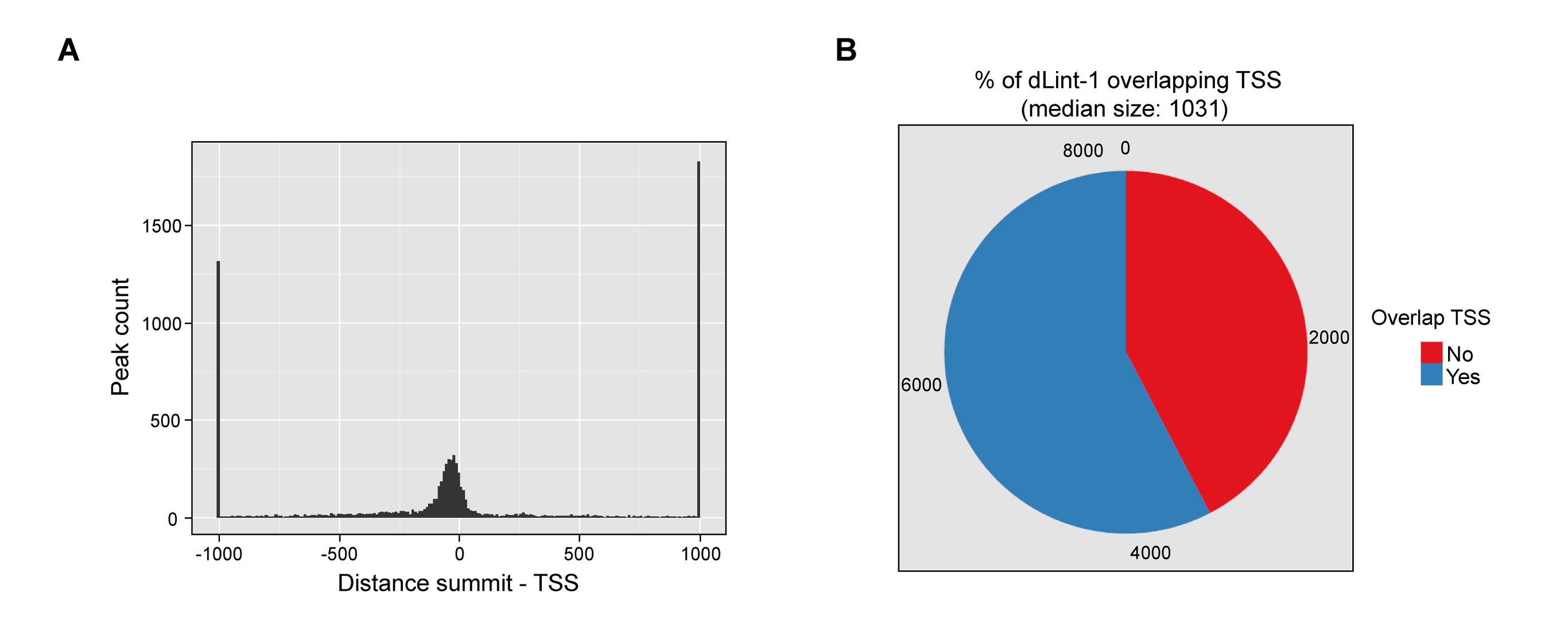

Supplement: Figure S5 — dLint-1 peaks cluster around TSSs. (A) Approximately 58% of dLint-1 peaks identified in S2 cells overlap with a known transcription start site (TSS). (B) Histogram depicting the distribution of distances from dLint1 peak summits (i.e. region of highest signal intensity) to the next TSS. Distances above 1000 bp were truncated to 1000 bp. (TIF) [file pgen.1002676.s005.tif]

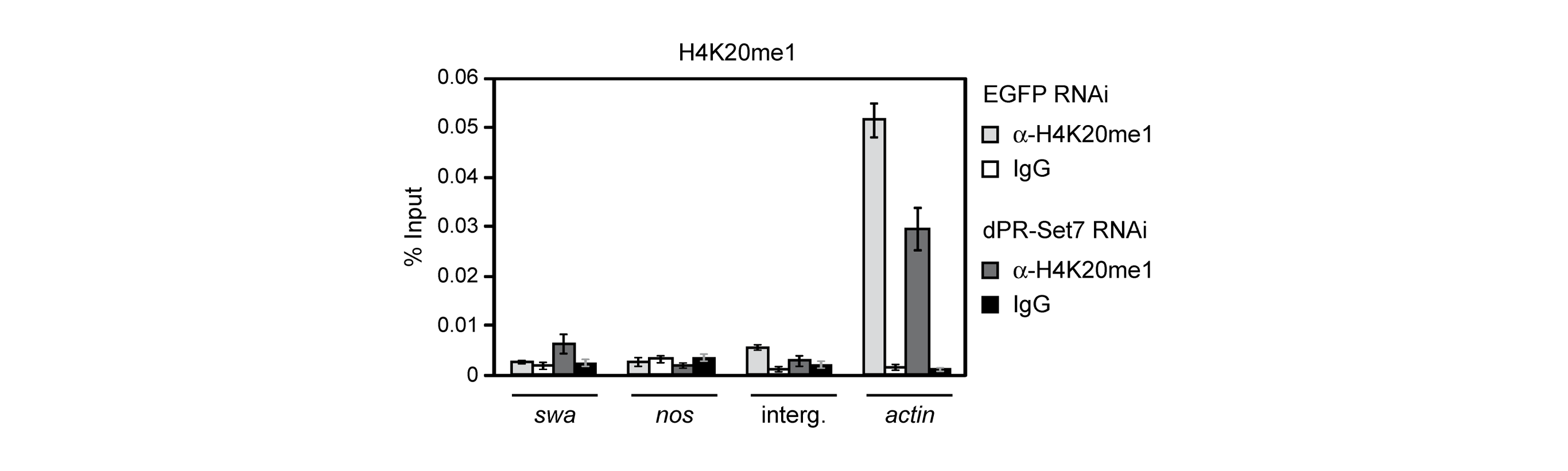

Supplement: Figure S6 — LINT target promoters are devoid of H4K20 mono-methylation. Chromatin from cells treated with RNAi against dPR-Set7 or EGFP (control) was precipitated with H4K20me1 or IgG antibodies as indicated. ChIP signals are shown for swa and nos promoter regions, an intergenic region and the actin gene as denoted below the panel. (TIF) [file pgen.1002676.s006.tif]
