# Supplementary material for: LINT, a Novel dL(3)mbt-Containing Complex, Represses Malignant Brain Tumour Signature Genes
Source: PLoS Genet. 2012 May 3;8(5):e1002676. doi: 10.1371/journal.pgen.1002676 (PMC3342951; doi:10.1371/journal.pgen.1002676)
Supplement: Table S1 — dL(3)mbt and dLint-1 bind to germline-specific MBTS and SWH target genes. Genes were visually inspected for dL(3)mbt peaks (Richter et al. 2011), dLint-1 peaks in Kc cells and dLint-1 peaks in S2 cells. +: at least one peak. (DOC) [file pgen.1002676.s007.doc]

**Table S1:**

| **Gene group** | **Genes** | **L(3)mbt** | **Lint-1 in Kc** | **Lint-1 in S2** |
| --- | --- | --- | --- | --- |
| **Germline-specific**  **MBTS genes** | Pxt | + | + | + |
|  | Fs(1)Yb | + | + | + |
|  | tej/CG8589 | + | + | + |
|  | zpg | + | + | + |
|  | bcgn | + | + | + |
|  | topi | + | + | + |
|  | TrxT | + | + | + |
|  | hdm | + | + | + |
|  | nos | + | + | + |
|  | ea | + | + | + |
|  | vas | + | + | + |
|  | CG9925 | + | + | + |
|  | krimp | + | + | + |
|  | dhd | + | + | + |
|  | CG7795 | + | + | + |
|  | piwi | + | + | + |
|  | RpS5b | + | + | + |
|  | gnu | + | + | + |
|  | CG32313 | + | + | + |
|  | mia | + | + | + |
|  | CG15930 | + | + | + |
|  | fus | + | + | + |
|  | stil | + | + | + |
|  | tor | + | + | - |
|  | γTub37C | + | (+) | + |
|  | tud | + | (+) | + |
|  | CG31755 | + | - | + |
|  | swa | + | - | + |
|  | Spn-E | + | - | + |
|  | bam | + | - | + |
|  | aub | - | + | + |
|  | cona | - | - | - |
| **SHW pathway** | diap1 | + | + | + |
|  | CycE | + | + | + |
|  | CycA | + | + | + |
|  | CycB | + | + | + |
|  | E2f | + | + | + |
|  | wg | + | + | + |
|  | ex | - | + | + |
|  | Ser | - | + | - |
|  | Mer | - | + | - |
|  | ban | - | - | + |
|  | fj | - | - | - |
