# Supplementary material for: LINT, a Novel dL(3)mbt-Containing Complex, Represses Malignant Brain Tumour Signature Genes
Source: PLoS Genet. 2012 May 3;8(5):e1002676. doi: 10.1371/journal.pgen.1002676 (PMC3342951; doi:10.1371/journal.pgen.1002676)
Supplement: Table S2 — dL(3)mbt and dLint-1 regulate germline-specific MBTS genes. Genes with a fold change ≥1.5 (adj. p≤0.05) were considered deregulated. (DOC) [file pgen.1002676.s008.doc]

**Table S**2:

| **Gene group** | **Genes** | **L(3)mbt RNAi** | | **Lint-1 RNAi** | |
| --- | --- | --- | --- | --- | --- |
|  |  | **Deregulation** | **Log2 FC** | **Deregulation** | **Log2 FC** |
| **Germline-specific**  **MBTS genes** | Pxt | up | 2.7 | - | 0.1 |
|  | Fs(1)Yb | - | 0.3 | up | 0.6 |
|  | tej/CG8589 | up | 6.8 | up | 6.0 |
|  | zpg | - | 0 | - | 0.1 |
|  | bcgn | - | 0.3 | up | 0.5 |
|  | topi | up | 0.6 | up | 0.9 |
|  | TrxT | up | 2.1 | up | 0.9 |
|  | hdm | up | 5.9 | up | 5.7 |
|  | nos | up | 5.6 | up | 3.9 |
|  | ea | up | 2.8 | up | 2.6 |
|  | vas | - | 0 | - | 0 |
|  | CG9925 | up | 3.7 | up | 1.8 |
|  | krimp | - | 0.1 | - | 0.1 |
|  | dhd | - | 0 | - | 0 |
|  | CG7795 | up | 2.0 | - | 0.3 |
|  | piwi | up | 7.7 | up | 6.6 |
|  | RpS5b | up | 6.5 | up | 4.4 |
|  | gnu | up | 1.1 | - | 0.4 |
|  | CG32313 | up | 6.0 | up | 5.4 |
|  | mia | up | 1.4 | up | 1.3 |
|  | CG15930 | - | 0.2 | - | 0 |
|  | fus | - | -0.5 | - | -0.2 |
|  | stil | - | 0 | - | 0 |
|  | tor | up | 2.7 | up | 1.5 |
|  | γTub37C | - | 0 | - | 0 |
|  | tud | down | -0.7 | - | -0.5 |
|  | CG31755 | - | 0.2 | - | 0.3 |
|  | swa | up | 4.8 | up | 4.4 |
|  | Spn-E | - | 0 | - | -0.1 |
|  | bam | up | 2.2 | up | 1.8 |
|  | aub | - | 0.4 | - | 0.4 |
|  | cona | up | 1.7 | up | 1.1 |
| **SHW pathway** | diap1 | - | 0 | - | 0 |
|  | CycE | - | 0 | down | -0.8 |
|  | CycA | - | 0 | - | -0.1 |
|  | CycB | - | 0.2 | - | 0 |
|  | E2f | - | 0 | - | -0.4 |
|  | wg | - | 0 | - | 0 |
|  | ex | - | -0.2 | down | -1.1 |
|  | Ser | - | 0.3 | - | 0.1 |
|  | Mer | - | -0.3 | - | 0 |
|  | ban |  | - |  | - |
|  | fj | - | 0 | - | 0 |
