# Supplementary material for: LINT, a Novel dL(3)mbt-Containing Complex, Represses Malignant Brain Tumour Signature Genes
Source: PLoS Genet. 2012 May 3;8(5):e1002676. doi: 10.1371/journal.pgen.1002676 (PMC3342951; doi:10.1371/journal.pgen.1002676)
Supplement: Table S3 — Top 50 genes repressed by Lint-1. (DOC) [file pgen.1002676.s009.doc]

**Table S**3:

| **Lint-1** | | **L(3)mbt** | | **Gene Symbol** | **Gene Title** | **Ovary/Testis-specific expression** |
| --- | --- | --- | --- | --- | --- | --- |
| **Rank** | **Log2 FC** | **Rank** | **Log2 FC** |
| 1 | 7.9 | 3 | 7.6 | CG14516 | CG14516 | Ovary |
| 2 | 7.2 | 2 | 8.8 | CG30296 | CG30296 |  |
| 3 | 6.5 | 4 | 7.6 | piwi | P-element induced wimpy testis | Ovary |
| 4 | 6.4 | 1 | 8.9 | CG11052 | CG11052 | Testis |
| 5 | 6.0 | 9 | 8.8 | CG17207 | CG17207 | Testis |
| 6 | 5.9 | 6 | 7.1 | CG5731 | CG5731 |  |
| 7 | 5.9 | 10 | 6.7 | CG8589 | anon-fast-evolving-1D11 | Ovary |
| 8 | 5.7 | 5 | 7.1 | mthl14 | methuselah-like 14 |  |
| 9 | 5.7 | 19 | 5.9 | hdm | hold'em |  |
| 10 | 5.7 | 14 | 6.1 | eIF4E-6 | eIF4E-6 |  |
| 11 | 5.7 | 18 | 5.9 | Acer | Angiotensin-converting enzyme-related |  |
| 12 | 5.6 | 24 | 5.4 | CG1623 | CG1623 |  |
| 13 | 5.4 | 16 | 5.9 | CG32313 | CG32313 | Testis |
| 14 | 5.4 | 20 | 5,9 | Rh4 | rhodopsin |  |
| 15 | 5.2 | 17 | 5.9 | CG9875 | CG9875 | Testis |
| 16 | 5.1 | 25 | 5.3 | CG8008 | CG8008 |  |
| 17 | 5.0 | 13 | 6.3 | skpB | skpB | Testis |
| 18 | 5.0 | 32 | 5.1 | Asph | Aspartyl beta-hydroxylase |  |
| 19 | 5.0 | 27 | 5.3 | CG11638 | CG11638 |  |
| 20 | 4.9 | 8 | 6.9 | CG30380 | CG30380 |  |
| 21 | 4.9 | 23 | 5.4 | CG5715 | CG5715 |  |
| 22 | 4.9 | 28 | 5.3 | GNBP3 | Gram-negative bacteria binding protein 3 |  |
| 23 | 4.7 | 31 | 5.1 | osm-6 | osm-6 | Testis |
| 24 | 4.7 | 36 | 5.0 | CG15737 | CG15737 | Ovary |
| 25 | 4.7 | 30 | 5.2 | Cyp6g1 | CYP6-like |  |
| 26 | 4.6 | 40 | 4.8 | Gbeta5 | Gbeta5 |  |
| 27 | 4.6 | 12 | 6.3 | dpr19 | dpr19 |  |
| 28 | 4.5 | 26 | 5.3 | TM4SF | Transmembrane 4 superfamily |  |
| 29 | 4.5 | 38 | 4.9 | tok | Tolkin |  |
| 30 | 4.5 | 7 | 7.0 | CG4596 | CG4596 |  |
| 31 | 4.4 | 46 | 4.5 | CG32436 | CG32436 | Testis |
| 32 | 4.4 | 11 | 6.4 | RpS5b | Ribosomal proteinS5b | Ovary/Testis |
| 33 | 4.4 | 39 | 4.8 | CG34232 | --- |  |
| 34 | 4.4 | 15 | 6.0 | Ef1alpha100E | elongation factor 1-alpha F2 |  |
| 35 | 4.3 | 41 | 4.8 | swa | swallow | Ovary |
| 36 | 4.3 | 65 | 4.0 | CG9542 | CG9542 |  |
| 37 | 4.3 | 45 | 4.5 | CG12698 | CG12698 | Testis |
| 38 | 4.3 | 51 | 4.3 | Hsp67Bc | Gene 3 |  |
| 39 | 4.1 | 43 | 4.8 | CG8046 | CG8046 |  |
| 40 | 4.1 | 79 | 3.8 | CG9961 | CG9961 | Testis |
| 41 | 4.1 | 52 | 4.3 | CG9427 | CG9427 |  |
| 42 | 4.0 | 47 | 4.5 | CG2887 | CG2887 | Testis |
| 43 | 4.0 | 84 | 3.7 | CG17625 | CG17625 | Testis |
| 44 | 3.9 | 22 | 5.6 | nos | nanos | Ovary |
| 45 | 3.9 | 42 | 4.8 | CG40303 | CG40303 |  |
| 46 | 3.9 | 90 | 3.5 | CG32187 | CG32187 |  |
| 47 | 3.8 | 21 | 5.7 | c(2)M | crossover suppressor | Ovary |
| 48 | 3.8 | 33 | 5.1 | CG34355 | CG34355 |  |
| 49 | 3.7 | 71 | 3.9 | CG17032 | CG17032 |  |
| 50 | 3.7 | 97 | 3.3 | CG6737 | CG6737 | Testis |
