# Supplementary material for: LINT, a Novel dL(3)mbt-Containing Complex, Represses Malignant Brain Tumour Signature Genes
Source: PLoS Genet. 2012 May 3;8(5):e1002676. doi: 10.1371/journal.pgen.1002676 (PMC3342951; doi:10.1371/journal.pgen.1002676)
Supplement: Table S4 — Primer sequences. (DOC) [file pgen.1002676.s010.doc]

**Table S4**

**Primer sequences.**

| **Primer for cloning** | | |
| --- | --- | --- |
| **Primer name** | **Sequence** | **Application** |
| FL-dL(3)mbt-fw | cgcTCTAGACCTGCCATTGTCGATGGCCAG | recombinant protein expression in S2 cells |
| FL-dL(3)mbt-rv | cgcGGATCCCTAAGAGGACGTGCGCAAGG |
| dLint-1-FL-fw | ttaaGCGGCCGCAGCAAGTACCATAAGGAGCGC |
| dLint-1-FL-rv | ggccCTCGAGGTCTTCTCCAACTTATCTTTTTCGC |
| dL(3)mbt-bac-fw | ggccGAATTCATGCTGCCATTGTCGATGGC | recombinant baculoviral protein expression in Sf9 cells |
| dL(3)mbt-FL-bac-rv | ttaaGGATCCCTATTTATCGTCATCGTCTTTGTAGTCAGAGGACGTGCGCAAGGG |
| dLint-1-bac-fw | ggccAGATCTATGAGCAAGTACCATAAGGAGC |
| dLint-1-bac-rv | ggccTCTAGACTACTTCTCCAACTTATCTTTTTC |
| dLint-1-ivt-fw | ccggTCTAGAAGCAAGTACCATAAGGAGCGCA | *in vitro* translation |
| dLint-1-ivt-rv | ccggTCTAGACTACTTCTCCAACTTATCTTTTTC |
| dL(3)mbt-lexA-fw | CCGGAATTCATGCTGCCATTGTCGATGGCC | recombinant protein expression of LexA fusions in S2 cells |
| dL(3)mbt-lexA-rv | AAAGCGGCCGCTAGAGGACGTGCGCAAGG |
| dLint-1-lexA-fw | GTAGTCGACGGGGTACCATGAGCAAGTACCATAAGGAGCGCA |
| dLint-1-lexA-rv | TTAAGCGGCCGCCCTTCTCCAACTTATCTTTTTC |
| **RNAi T7 primer for dsRNA synthesis** | | |
| EGFP-RNAi-fw | gaattaatacgactcactatagggaGAGCTGGACGGCGACGTAA | |
| EGFP-RNAi-rv | gaattaatacgactcactatagggagACTTGTACAGCTCGTCCATG | |
| dL(3)mbt-RNAi-fw | taatacgactcactatagggGTTGGTTTGGGTGCTGTCTT | |
| dL(3)mbt-RNAi-rv | taatacgactcactatagggGCGTCTAAAGTTCAGCCAGG | |
| dLint-1-RNAi-fw | taatacgactcactatagggATGAAAGGGTCGCTGGATT | |
| dLint-1-RNAi-rv | taatacgactcactatagggGCTCGGCACTGGAATCAT | |
| dRpd3- RNAi-fw | taatacgactcactatagggCGACGGCGTCTAATACCAAT | |
| dRpd3- RNAi-rv | taatacgactcactatagggCCGCCCACTGATTACTGATT | |
| dCoREST-RNAi-fw | taatacgactcactatagggCATTCGCTCAGTTTTCTGACG | |
| dCoREST-RNAi-rv | taatacgactcactatagggCCACCGAAATGTACTCCTCC | |
| dLsd1-RNAi-fw | taatacgactcactatagggAAAGAAACGTCAATCACCCG | |
| dLsd1-RNAi-rv | taatacgactcactatagggCCTCTTCGTTGGGTGTCATT | |
| dPR-Set7-RNAi-fw | taatacgactcactatagggATGGTCTCCAAGTACGCCAC | |
| dPR-Set7-RNAi-rv | taatacgactcactatagggCCAAAAACCAGTTTAGCCCA | |
| G9a-RNAi-fw | taatacgactcactatagggAAACCAAGTGTTACTTTGAGAG | |
| G9a-RNAi-rv | taatacgactcactatagggTGTACAAAATATGCCACATCCT | |
| Pc-RNAi-fw | taatacgactcactatagggGAAGCCATAAACACAACGCC | |
| Pc-RNAi-rv | taatacgactcactatagggACATTTGTTTGGGTCGAAGC | |
| **RT-qPCR primer** | | |
| gapdh1-fw | gagcaaggactaaactagccaaa | |
| gapdh1-rv | caacagtgattcccgacca | |
| l(3)mbt-RT-fw | tttctggcaccacatttctg | |
| l(3)mbt-RT-rv | ctctccttctgcgtactctgc | |
| lint-1-RT-fw | gcaggagcagcaaagacg | |
| lint-1-RT-rv | ctcaaagaggccgaggaac | |
| piwi-RT-fw | cagaagtacaaggccggataa | |
| piwi-RT-rv | tttgccaatcagcgttttct | |
| nos-RT-fw | gcgcgatccttgaaaatct | |
| nos-RT-rv | gcgaactcctgcatcacat | |
| swa-RT-fw | gctgatggcagcggtagt | |
| swa-RT-rv | ggctggtttccgagttgtt | |
| ea-RT-fw | cgaaaatgctaaagccatcg | |
| ea-RT-rv | cgttggggaagtagaactgg | |
| tor-RT-fw | gcctgcagaactttttacgtg | |
| tor-RT-rv | tgtccacgttctgttcaagg | |
| bam-RT-fw | gagcaatgcggacaagttc | |
| bam-RT-rv | tagcggtgctccagatcc | |
| zpg-RT-fw | cgtcttctgcgaaatactcaattt | |
| zpg-RT-rv | cactggttatagtcgccattgt | |
| tud-RT-fw | aagaagcctttgctgctttg | |
| tud-RT-rv | cctcgttcggctgagtagtt | |
| **ChIP-qPCR primer** | | |
| swa-b-fw | TTTCGCCAAAGGCAATAGATG | |
| swa-b-rv | GGCGAGATCGAGGAGTATG | |
| swa-c-fw | TGCAGAGAAGCAATTTCACG | |
| swa-c-rv | CAATTAAATATGGCAGCGAATTG | |
| swa-d-fw | GTTAGCACCGAAGCTGATGG | |
| swa-d-rv | GCTGGTTTCCGAGTTGTTGG | |
| nos-b-fw | GTCATCGTTTCCGAAAGCTC | |
| nos-b-rv | AGGTATGGAGCTGCACAAGG | |
| nos-c-fw | CATGTGATGTTGTCACAGTGC | |
| nos-c-rv | ACTTGCTAAGAATATGTGCCAC | |
| nos-d-fw | ATGTCCTACGGGAGTGCTC | |
| nos-d-rv | CACACGTTGTTCAGATGCTC | |
| piwi-a-fw | CTGGTTGACCAACGATTGC | |
| piwi-a-rv | CGATAACTGCAACCTGATCG | |
| piwi-b-fw | CTTACCAATGCTTGGATCGAC | |
| piwi-b-rv | GGAACCAATCCGAGGAGCTG | |
| piwi-c-fw | TAAGGCTCTTGCAACGATTG | |
| piwi-c-rv | TTTTTTGGTACTTCGAGCTTTG | |
| piwi-d-fw | CCGTGGGGTGACCAATATG | |
| piwi-d-rv | GATCCCAGAAGGTTAGCATG | |
| tok-b-fw | GCGACGCTTGGTGAGAATC’ | |
| tok-b-rv | GCAGAAAACTCTGCCGTAG | |
| tok-c-fw | TTTGGGAAATTTGCCTTACG | |
| tok-c-rv | AACCCTGGCACAAACGTATC | |
| tok-d-fw | TCTGATTTCGCTTTGTGTCG | |
| tok-d-rv | GGCCAGACAAGATGAGTTGG | |
| crb-b-fw | CTAAGCGCCCAATGCTACTC | |
| crb-b-rv | TTTTACCAGCCAGGAATTGG | |
| crb-c-fw | TGCTGCTTGCAGTTCAAAAG | |
| crb-c-rv | GCACGCGACACTTTCTAGC | |
| crb-d-fw | CCTCATTTGTCTGCATTTTCA | |
| crb-d-rv | CACAATCCGTTGGAAAAAGG | |
| ea-b-fw | GAATGAAGGCATTGCGACTC | |
| ea-b-rv | CCCACGGAATACGGGATAC | |
| ea-c-fw | AGGCGGGGATTAAATAATGG | |
| ea-c-rv | TGGCGAGTCTCCAAATCAG | |
| ea-d-fw | GTGCCAGAACGTCTACAGC | |
| ea-d-rv | GGAGTCGGTCCAAATGACAC | |
| spn-E-b-fw | TCTGTTTCTAATAACTGACCAGCAA | |
| spnE-b-rv | TTCCACGTTGTATTCGACAGA | |
| spnE-c-fw | GCTGGGTTTCTTTCGATTACC | |
| spnE-c-rv | TTTTTCTCACAGTCTTTCTGGATT | |
| spnE-d-fw | TTCTGCATCAAGGCACTCTG | |
| spnE-d-rv | GCTCTAGATCTGCGCAAGGA | |
| tud-b-fw | TTAACGAATGGCTTTCCTCCT | |
| tud-b-rv | CGGATTTCAGTAGCGTCCTC | |
| tud-c-fw | CACGGCGACGTACCTAAAAT | |
| tud-c-rv | TTCCACAAGACAACGAAACG | |
| tud-d-fw | TGGAAGGATAATCCGCAGTC | |
| tud-d-rv | CGGCAGATGTTTAGGCGTAT | |
| actin5C-fw | accggtaTCGTTCTGGACTC | |
| actin5C-rv | CGGTCAGGATCTTCATCAGG | |
| interg-2R-fw | tgctgactgccatcaaattc | |
| interg-2R-rv | tacttgctgtgacggctttg | |
